# Fluorinated Arylarsonate-Containing Polyoxomolybdates: pH-Dependent Formation of Mo_6_ vs Mo_12_ Species and Their Solution Properties

**DOI:** 10.1021/acs.inorgchem.4c02951

**Published:** 2024-09-26

**Authors:** Arun Pal, Saurav Bhattacharya, Xiang Ma, Ahmad Ben Kiran, Cristian Silvestru, Ulrich Kortz

**Affiliations:** † School of Science, Constructor University, Campus Ring 1, Bremen 28759, Germany; ‡ Department of Chemistry, BITS Pilani K. K. Birla Goa Campus, Zuarinagar 403726 Goa, India; § Fujian Provincial Key Laboratory of Advanced Inorganic Oxygenated Materials, College of Chemistry, Fuzhou University, Fuzhou 350108 Fujian, China; ∥ Department of Chemistry, Supramolecular Organic and Organometallic Chemistry Centre (SOOMCC), Faculty of Chemistry and Chemical Engineering, Babeş-Bolyai University, 11 Arany Janos, Cluj-Napoca 400028, Romania

## Abstract

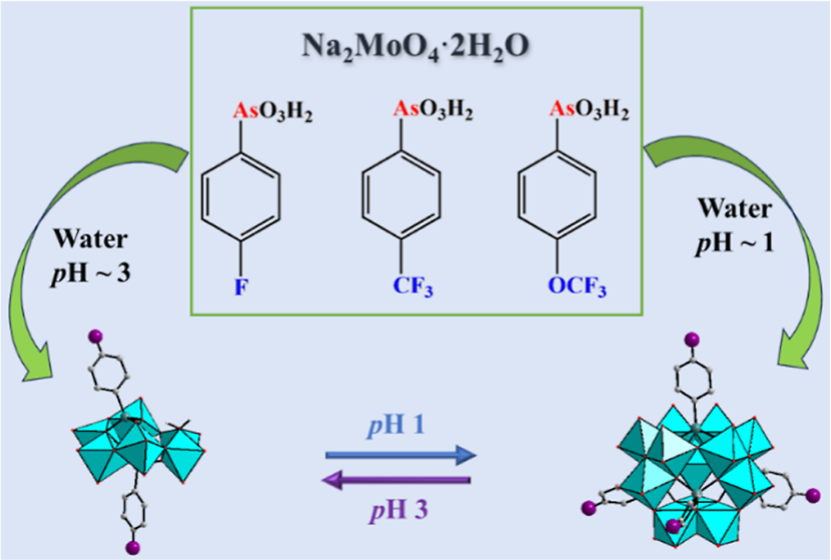

We report on the synthesis and structural characterization of six novel arylarsonate-containing polyoxomolybdates with fluorinated-functionalities in the *para* position of the phenyl ring. The reaction of the various arylarsonic acids, RAsO_3_H_2_ [*R* = 4-F–C_6_H_4_ (H_2_**L**_**F**_), 4-F_3_C–C_6_H_4_ (H_2_**L**_**CF3**_), 4-F_3_CO–C_6_H_4_ (H_2_**L**_**OCF3**_)] with Na_2_MoO_4_·2H_2_O in aqueous pH 3 solution resulted in the heteropoly-6-molybdates [{(4-F–C_6_H_4_)As}_2_Mo_6_O_24_(H_2_O)]^4–^ (**1**), [{(4-F_3_C–C_6_H_4_)As}_2_Mo_6_O_24_]^4–^ (**2**) and [{(4-F_3_CO–C_6_H_4_)As}_2_Mo_6_O_24_(H_2_O)]^4–^ (**3**), which were isolated as guanidinium salts. When the reaction was performed in aqueous pH 1 solution the inverted-Keggin type heteropoly-12-molydates [{(4-F–C_6_H_4_)As}_4_Mo_12_O_46_]^4–^ (**4**), [{(4-F_3_C–C_6_H_4_)As}_4_Mo_12_O_46_]^4–^ (**5**) and [{(4-F_3_CO–C_6_H_4_)As}_4_Mo_12_O_46_]^4–^ (**6**), were obtained and isolated as sodium salts. The 6-molybdates **1**–**3** and the 12-molybdates **4**–**6** can be easily interconverted reversibly in solution as a function of pH (3 vs 1). Polyanions **1** and **3** are isostructural and they exhibit a bent hexamolybdate ring, whereas the ring is flat for **2**. The inverted-Keggin polyanions **4**–**6** are isostructural and the metal-oxo core is capped by four arylarsonate groups. All six polyanions have been characterized in the solid state by single-crystal X-ray diffraction, Fourier transform infrared spectroscopy, and hermogravimetric analysis as well as in solution by multinuclear NMR (^1^H, ^19^F). The synthetic procedures for the arsonic acids (4-F_3_C–C_6_H_4_)AsO_3_H_2_ (H_2_**L**_**CF3**_) and (4-F_3_CO–C_6_H_4_)AsO_3_H_2_ (H_2_**L**_**OCF3**_) are reported for the first time.

## Abstract 2

Fluorinated arylarsonate-containing polyoxo-6-molybdates and polyoxo-12-molybdates have been synthesized and characterized in the solid state and in solution. The Mo_6_ and Mo_12_ polyanions are interconverted reversibly as a function of *p*H (3 vs 1).

## 1 Introduction

Polyoxometalates (POMs) are discrete, anionic and soluble metal-oxo clusters of early transition metal ions in high oxidation states (e.g., W^VI^, Mo^VI^, V^V^) and they exhibit a very large range of shapes, sizes and compositions.^1^ POMs attract significant attention across various research fields, such as catalysis, magnetism, and electronics, as they show exceptional diversity in structure and composition, thermal stability, and capability to undergo multielectron redox reactions under mild conditions.^2^ In addition, POMs have been demonstrated to possess significant potential as antitumor, anticancer, antibacterial, antimicrobial, and antidiabetic agents in both in vitro and in vivo studies.^3^ However, certain challenges remain in terms of achieving target specificity for these applications. An elegant solution to address this challenge involves covalent grafting of organic groups onto the POMs.^4^ This approach not only enhances selectivity toward the target but also improves the stability at physiological pH levels.^5^ Previous research in this area has yielded a substantial body of knowledge regarding the grafting of organic groups and ligands onto POMs, with extensive reports on POMs containing phosphites, phosphonates, amino acids, and other organic moieties.^4,6^ Several organoarsonate-stabilized polyoxomolybdates have been reported over the years. The hexanuclear, cyclic polyanion family [(RAsO_3_)_2_Mo_6_O_18_]^4–^ (*R* = CH_3_, Ph, 4-NH_2_–C_6_H_4_)^7a^ was reported by Pope’s group in 1976 and two years later Matsumoto prepared [(PhAs)_2_Mo_6_O_25_H_2_]^4–^.^7b^ Liu’s group reported the n-propyl derivative (*R* = *n*-C_3_H_7_)^8^ in 1988, and in 2021 Kortz’s group reported a bromo-phenyl and an azido-phenyl derivative (*R* = 4-Br–C_6_H_4_, 4-N_3_–C_6_H_4_).^9^ In 1996 Zubieta’s group reported the solid state structure of the tetranuclear polyanion [(RAsO_3_)_4_Mo_4_O_10_]^4–^.^10^ Several members of the dodecanuclear polyanion family [(RAsO_3_)_4_Mo_12_O_34_]^4–^ (*R* = Ph, 4-Me–C_6_H_4_, 4-NH_2_–C_6_H_4_, 4-OH–C_6_H_4_, 4-HOOC–C_6_H_4_, 3-NO_2_–4-OH–C_6_H_3_, 4-NC–C_6_H_4_, C_2_H_4_OH, 4-N_3_C_2_H_2_–C_6_H_4_) were reported by various groups.^11^

Fluorine plays a crucial role in biomedical and pharmaceutical applications due to its unique properties, such as improving metabolic stability and enhancing membrane permeation.^12^ Around 20% of all pharmaceutical products contain at least one fluorine atom,^13^ and drugs such as Liptor (atorvastatin calcium) by Pfizer/Astellas are among the blockbusters on the market.^14^ While most organoarsenic compounds are toxic for most biological purposes, certain arylarsonic acids, such as (3-nitro-4-hydroxy-phenyl)arsonic acid, have been utilized in veterinary formulations to prevent infections and promote growth factors.^15^ The anti-infective properties of arylarsonates are well-documented,^16^ but further research is needed to explore and maximize their effects.

This work aims at the synthesis of novel fluorine-containing polyoxomolybdates using known and novel fluorinated organoarsonate hetero groups.

## 2 Experimental Section

### 2.1 Materials and Reagents

**Scheme 1:**
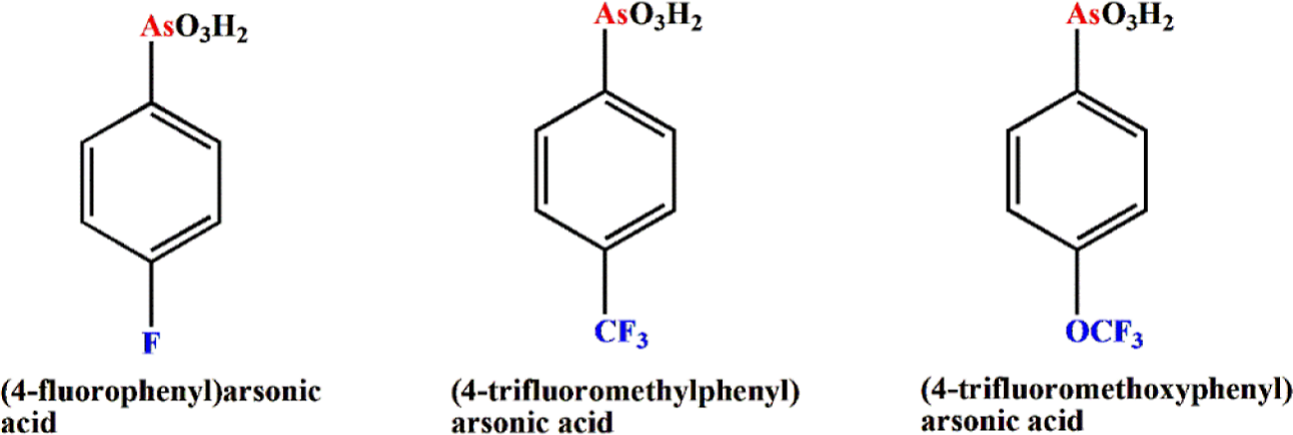
Structure of Fluorinated Arylarsonic Acids Used as Hetero Groups in This Study

All commercially available chemicals were received as reagent-grade quality, with no further purification before use. The (4-fluorophenyl)arsonic acid (4-F–C_6_H_4_)AsO_3_H_2_ (H_2_**L**_**F**_) (see Scheme 1, left) was prepared according to a known literature procedure.^17^ On the other hand, we prepared new arylarsonic acids, i.e. (4-trifluoromethylphenyl)arsonic acid (4-F_3_C–C_6_H_4_)AsO_3_H_2_ (H_2_**L**_**CF3**_), and (4-trifluoromethoxyphenyl)arsonic acid (4-F_3_CO–C_6_H_4_)AsO_3_H_2_ (H_2_**L**_**OCF3**_), respectively (see Scheme 1, middle and right), using an adapted method reported for the synthesis of (4–Br–C_6_H_4_)AsO_3_H_2_.^9^

### 2.2 Physical Measurements

The multinuclear NMR spectra (^1^H, ^13^C, and ^19^F) were performed at room temperature using a JEOL ECX 400 MHz instrument. The recorded chemical shifts are presented relative to Si(CH_3_)_4_ (for ^1^H and ^13^C) and CFCl_3_ (for ^19^F) references. The FT-IR spectra were acquired on a Nicolet-Avatar 370 spectrometer from 4000 to 400 cm^–1^ using KBr disks. Thermogravimetric analyses (TGA) were performed on a TA Instruments Q600 apparatus, using a nitrogen gas flow and heating rate of 5 °C per minute from room temperature to 500 °C. Elemental analyses were performed by Analytische Laboratorien in Lindlar, Germany. Sodium (Na) was determined in-house by atomic absorption spectroscopy using a Varian SpectrAA 220 AA instrument.

### 2.3 X-Ray Crystallography

Single crystals of the salts of polyanions **1**, **2** and **4**–**6** were mounted on a Hampton cryoloop utilizing paratone oil. Subsequent data collections were conducted using a Bruker Kappa X8 APEX II CCD diffractometer, employing a sealed molybdenum tube and graphite monochromator, with Kα radiation (λ = 0.71073 Å) at a temperature of 100 K. The data collection for polyanion **3** was conducted on a Rigaku Oxford Diffraction XtaLAB Synergy instrument, employing a sealed copper tube and graphite monochromator, with Kα radiation (λ = 1.54184 Å) at a temperature of 100 K. For absorption corrections, an empirical method was employed through the utilization of the SADABS program.^18^ The SHELX software package from Bruker facilitated the resolution and refinement of the structures.^19^ The structural solutions were initially derived through direct methods, followed by refinement via the full-matrix least-squares approach [Σw (|*F*_o_|^2^ – |*F*_c_|^2^)^2^], encompassing anisotropic thermal parameters for all heavy atoms included within the model. The inclusion of hydrogen atoms positioned on carbon atoms was realized through calculated coordinates, integrated within the refinement process, and associated with their respective parent atoms. Not all sodium counter cations could be precisely identified via Fourier electron density due to crystallographic disorder. Particularly challenging reflections were excluded employing the SQUEEZE command within PLATON.^20^ The true elemental composition of bulk material for all samples was established by elemental analysis and hence the actual number of counter cations and crystal water molecules is also based on elemental analysis. The resulting formula units are used throughout the manuscript and reflected in the CIF file to ensure overall consistency. The crystallography data and structure refinement parameters for all six compounds are shown in Table 1. Cambridge crystallographic data files CCDC 2366189–2366194 contain the supplementary crystallographic data for this paper. These data can be obtained free of charge from The Cambridge Crystallographic Data Center via www.ccdc.cam.ac.uk/.

**Table 1:** Crystal Data and Structure Refinements for Polyanions **1**–**6**

compound	**Gua-1**	**Gua-2**	**Gua-3**
empirical formula^a^	C_16_H_34_As_2_F_2_Mo_6_N_12_O_25_ (C_16_H_42_As_2_F_2_Mo_6_N_12_O_29_)	C_18_H_32_As_2_F_6_Mo_6_N_12_O_24_ (same formula as above)	C_17_H_28_As_2_F_6_Mo_6_N_9_O_27_ (C_18_H_42_As_2_F_6_Mo_6_N_12_O_31_)
formula weight^a^	1558.03 (1630.05)	1640.03	1629.96 (1762.06)
temperature (K)	100 (2)	100 (2)	100 (2)
radiation	Mo–Kα	Mo–Kα	Cu–Kα
wavelength (λ)	0.71073 Å	0.71073 Å	1.54184 Å
crystal system	monoclinic	monoclinic	triclinic
space group	*Cc*	*P*2_1_/*n*	*P*-1
*a* (Å)	15.8446 (16)	10.4531 (7)	13.2653 (3)
*b* (Å)	21.2623 (16)	18.2949 (12)	18.8924 (3)
*c* (Å)	15.5483 (13)	12.2437 (8)	21.3137 (4)
α (°)	90	90	107.3860 (10)
β (°)	95.315 (3)	106.771 (2)	101.929 (2)
γ (°)	90	90	90.3100 (10)
volume (Å^3^)	5215.6 (8)	2241.9 (3)	4974.45 (17)
*Z*	4	2	4
density (calculated) [Mg/m^3^]	1.984	2.430	2.176
absorption coefficient (mm^–1^)	2.745	3.211	14.513
*F*(000)	3000	1576	3124
refl. used [*I* > 2σ (*I*)]	8460	5114	18,650
independent reflections	9282	5597	21,129
*R*_int_	0.0667	0.0429	0.1108
refinement method	full-matrix least-squares on *F*^2^	full-matrix least-squares on *F*^2^	full-matrix least-squares on *F*^2^
GOF	1.060	1.054	1.041
final *R*_1_^b^, *wR*_2_^c^ [*I* > 2σ (*I*)]	*R*_1_ = 0.0402 *wR*_2_ = 0.1021	*R*_1_ = 0.0184 *wR*_2_ = 0.0421	*R*_1_ = 0.0886 *wR*_2_ = 0.2261
*R*_1_^b^, *wR*_2_^c^ (all data)	*R*_1_ = 0.0449 *wR*_2_ = 0.1047	*R*_1_ = 0.0222 *wR*_2_ = 0.0434	*R*_1_ = 0.0968 *wR*_2_ = 0.2332

	**Na-4**	**Na-5**	**Na-6**
empirical formula^a^	C_24_H_16_As_4_F_4_Mo_12_O_46_ (C_24_H_46_As_4_F_4_Mo_12_Na_4_O_61_)	C_28_H_16_As_4_F_12_Mo_12_O_46_ (C_28_H_72_As_4_F_12_Mo_12_Na_2_O_73_)	C_28_H_16_As_4_F_12_Mo_12_O_50_ (C_28_H_56_As_4_F_12_Mo_12_Na_2_O_69_)
formula weight^a^	2567.33 (2929.5)	2767.37 (3301.75)	2831.37 (3221.63)
temperature (K)	100 (2)	100 (2)	100 (2)
radiation	Mo–Kα	Mo–Kα	Mo–Kα
wavelength (λ)	0.71073 Å	0.71073 Å	0.71073 Å
crystal system	monoclinic	monoclinic	tetragonal
space group	*C*2/*c*	*C*2/*m*	*I*-4
*a* (Å)	24.003 (2)	25.710 (4)	20.012 (3)
*b* (Å)	24.491 (2)	25.644 (4)	20.012 (3)
*c* (Å)	24.149 (2)	15.005 (2)	11.5593 (19)
α (°)	90	90	90
β (°)	90.515 (3)	115.882 (5)	90
γ (°)	90	90	90
volume (Å^3^)	14,196 (2)	8900 (2)	4629.4 (16)
*Z*	8	4	2
density (calculated) [Mg/m^3^]	2.403	2.065	2.031
absorption coefficient (mm^–1^)	3.994	3.205	3.087
*F* (000)	9600	5184	2656
refl. used [*I* > 2σ (*I*)]	8293	5290	3359
independent reflections	13,554	8886	3933
*R*_int_	0.1243	0.1713	0.1101
refinement method	full-matrix least-squares on *F*^2^	full-matrix least-squares on *F*^2^	full-matrix least-squares on *F*^2^
GOF	0.973	1.042	1.064
final *R*_1_^b^, *wR*_2_^c^ [*I* > 2σ (*I*)]	*R*_1_ = 0.0647 *wR*_2_ = 0.1712	*R*_1_ = 0.0885 *wR*_2_ = 0.2432	*R*_1_ = 0.0476 *wR*_2_ = 0.1326
*R*_1_^b^, *wR*_2_^c^ (all data)	*R*_1_ = 0.0937 *wR*_2_ = 0.1832	*R*_1_ = 0.1384 *wR*_2_ = 0.2929	*R*_1_ = 0.0596 *wR*_2_ = 0.1409

a The entries in brackets are the actual formula units and weights as obtained from elemental analysis on bulk samples.

b *R*_1_ = Σ||*F*_o_| – |*F*_c_||/Σ|*F*_o_|.

c *wR*_2_ = [Σw (*F*_o_^2^ – *F*_c_^2^)^2^/Σw (*F*_o_^2^)^2^]^1/2^.

### 2.4 Synthetic Procedures

#### 2.4.1 Synthesis of (4-fluorophenyl)arsonic Acid, (4-F–C_6_H_4_)AsO_3_H_2_ (H_2_**L**_**F**_)

4-Fluoroaniline (1.92 mL, 20.0 mmol) was dissolved in 25 mL of 2 M HCl_aq_ at 0 °C and kept at this temperature for 10 min. To this solution, sodium nitrite (1.68 g, 24.3 mmol) in 5 mL of water was added dropwise and the mixture was stirred for additional 15 min at 0 °C, resulting in solution (**1**). In a separate container, solid Na_2_CO_3_ (5.3 g, 50 mmol) and As_2_O_3_ (2.5 g, 12.6 mmol) were added to 25 mL of water at 80 °C. When these solid starting materials were completely dissolved, solid CuSO_4_·5H_2_O (0.11 g, 0.44 mmol) was added. The resultant blue solution (**2**) was cooled to 0 °C. Next, solution (**1**) was gradually added dropwise to solution (**2**) at 0 °C over the course of 1 h while continuously stirring at a high speed. As the reaction progressed, a strong effervescence occurred due to the release of nitrogen gas (N_2_) from the intermediate diazonium salt. Once the addition was complete, the reaction mixture was left overnight at room temperature, under constant stirring. This reaction mixture gradually turned reddish during this period. Subsequently, the mixture was filtered off, and the yellowish filtrate was collected. The pH of the filtrate was adjusted to 7 using concentrated HCl_aq_ and then filtered again. Further, the pH was lowered to 1 with conc. HCl_aq_ and filtered once more. Then the filtrate was concentrated by boiling the solution down to half of the initial volume. Upon cooling down, (4-F–C_6_H_4_)AsO_3_H_2_ (H_2_**L**_**F**_) was formed as a white crystalline product; the solution was kept in a fridge overnight to obtain a higher yield. Afterward, the compound was filtered under vacuum, washed with cold H_2_O and dried open to the air at room temperature. Yield: 2.5 g (57%). ^1^H NMR (DMSO-*d*_6_, 400 MHz, ppm): δ 7.42 (m, 2H, Ar), 7.79 (m, 2H, Ar) (Figure S1). ^19^F NMR (DMSO-*d*_6_, 376.5 MHz, ppm): δ –105.7 (Figure S2). ^13^C{^1^H} NMR (DMSO-*d*_6_, 100 MHz, ppm): δ 116.8 (d, ^2^J_CF_ = 22.0 Hz), 130.0 (d, ^4^J_CF_ = 3.3 Hz), 133.0 (d, ^3^J_CF_ = 9.1 Hz), 164.7 (d, ^1^J_CF_ = 250.8 Hz) (Figure S3). ^1^H NMR (D_2_O/H_2_O, 400 MHz, ppm): δ 7.23 (m, 2H, Ar), 7.71 (m, 2H, Ar). ^19^F NMR (D_2_O/H_2_O, 376.5 MHz, ppm): δ –103.3. FT-IR (2% KBr disk, υ/cm^–1^): 2849 (br), 2349 (br), 1905 (m), 1781 (w), 1653 (sh), 1588 (s), 1491 (s), 1462 (sh), 1396 (s), 1301 (s), 1230 (s), 1164 (s), 1091 (s), 1008 (m), 955 (sh), 907 (s), 829 (w), 793 (w), 750 (w), 628 (sh), 607 (s), 511 (s). Abbreviations: s = strong, m = medium, w = weak, br = broad, sh = shoulder.

#### 2.4.2 Synthesis of (4-trifluoromethylphenyl)arsonic Acid, (4-F_3_C–C_6_H_4_)AsO_3_H_2_ (H_2_**L**_**CF3**_)

The compound (4-F_3_C–C_6_H_4_)AsO_3_H_2_ (H_2_**L**_**CF3**_) was synthesized using the same procedure as that for H_2_**L**_**F**_, but by using 2.5 mL (20.0 mmol) of 4-(trifluoromethyl)aniline instead of 4-fluoroaniline. Yield: 2.5 g (47%). Elemental analysis calculated for H_2_**L**_**CF3**_: As, 27.7; F, 21.11; C, 31.13; H, 2.2. Found: As, 26.0; F, 22.52; C, 33.35; H, 2.3. ^1^H NMR (DMSO-*d*_6_, 400 MHz, ppm): δ 7.95 (s, 4H, Ar) (Figure S4). ^19^F NMR (DMSO-*d*_6_, 376.5 MHz, ppm): δ –61.8 (Figure S5). ^13^C{^1^H} NMR (DMSO-*d*_6_, 100 MHz, ppm): δ 123.7 (q, ^1^J_CF_ = 272.9 Hz), 126.4 (q, ^3^J_CF_ = 3.8 Hz), 131.3 (s), 132.8 (q, ^2^J_CF_ = 32.1 Hz), 138.6 (s) (Figure S6). ^1^H NMR (D_2_O/H_2_O, 400 MHz, ppm): δ 7.88 (m, 4H, Ar). ^19^F NMR (D_2_O/H_2_O, 376.5 MHz, ppm): δ –63.3. FT-IR (2% KBr disk, υ/cm^–1^): 3436 (br), 2911 (br), 2387 (br), 1931 (w), 1789 (w), 1606 (s), 1504 (w), 1404 (s), 1331 (s), 1291 (sh), 1225 (m), 1186 (s), 1153 (sh),1132 (s), 1109 (s), 1086 (s), 1023 (s), 957 (s), 900 (s), 866 (s), 832 (s), 767 (s), 726 (sh), 693 (s), 596 (s), 499 (s), 439 (s).

#### 2.4.3 Synthesis of (4-trifluoromethoxyphenyl)arsonic Acid, (4-F_3_CO–C_6_H_4_)AsO_3_H_2_ (H_2_**L**_**OCF3**_)

The compound (F_3_CO–C_6_H_4_AsO_3_H_2_) (H_2_**L**_**OCF3**_) was synthesized using the same procedure as that for H_2_**L**_**F**_, but by using 2.7 mL (20.0 mmol) of 4-(trifluoromethoxy)aniline instead of 4-fluoroaniline. Yield: 2.6 g (46%). Elemental analysis calculated for H_2_**L**_**OCF3**_: As, 26.2; F, 19.93; C, 29.39; H, 2.11. Found: As, 25.7; F, 19.85; C, 30.13; H, 2.14. ^1^H NMR (DMSO-*d*_6_, 400 MHz, ppm): δ 7.59 (m, 2H, Ar), 7.87 (m, 2H, Ar) (Figure S7). ^19^F NMR (DMSO-*d*_6_, 376.5 MHz, ppm): δ –56.6 (Figure S8). ^13^C{^1^H} NMR (DMSO-*d*_6_, 100 MHz, ppm): δ 119.0 (q, ^1^J_CF_ = 257.8 Hz), 120.9 (s), 131.8 (s), 132.1 (s), 150.5 (s) (Figure S9). ^1^H NMR (D_2_O/H_2_O, 400 MHz, ppm): δ 7.46 (d, 2H, Ar), 7.82 (d, 2H, Ar). ^19^F NMR (D_2_O/H_2_O, 376.5 MHz, ppm): δ –57.5. FT-IR (2% KBr disk, υ/cm^–1^): 3429 (br), 2832 (br), 2315 (br), 1597 (s), 1584 (s), 1500 (s), 1470 (sh), 1411 (m), 1383(sh), 1325 (s), 1306 (s), 1274 (s), 1208 (s), 1179 (s), 1121 (sh), 1099 (s), 1020 (s), 929 (s), 890 (s), 833 (s), 791 (s), 773 (m), 757 (s), 670 (s), 622 (w), 521 (s), 442 (sh), 423 (s).

#### 2.4.4 Synthesis of [(NH_2_)_3_C]_4_[{(4-F–C_6_H_4_)As}_2_Mo_6_O_24_(H_2_O)]·4H_2_O (**Gua-1**)

0.022 g (0.10 mmol) of (4-fluorophenyl)arsonic acid (H_2_**L**_**F**_) and 0.073 g (0.30 mmol) of Na_2_MoO_4_·2H_2_O were mixed in 5 mL of H_2_O and stirred at room temperature for a few minutes. After complete dissolution, the pH of the solution was adjusted between 2.5–3.5 with 2 M H_2_SO_4(aq)_. The solution was stirred at ∼80 °C for 1 h and then 0.5 mL of 1 M guanidinium chloride solution was added, with continued stirring for another 15 min. Then, the solution was allowed to cool down to room temperature, filtered and kept for crystallization. Colorless crystals of **Gua-1** were obtained the next day. Yield: 60 mg (74% based on Mo). Polyanion **1** is obtained in 100% reaction yield at room temperature, but the isolated yield of the guanidinium salt is higher when the reaction solution is heated at 80 °C. We do not know the reason for this, but we decided to report the latter condition in the experimental procedure above. Elemental analysis calculated for **Gua-1**: Mo, 35.3; As, 9.19; F, 2.3; C, 11.79; H, 2.60; N, 10.3. Found: Mo, 35.0; As, 9.44; F, 3.0; C, 11.94; H, 2.85; N, 10.2. ^1^H NMR (D_2_O/H_2_O, 400 MHz, ppm): δ 7.23 (m, 2H, Ar), 8.15 (m, 2H, Ar) (Figure S10). ^19^F NMR (D_2_O/H_2_O, 376.5 MHz, ppm): δ –106.0 (Figure 2, black). FT-IR (2% KBr disk, cm^–1^): 3404 (br), 3178 (w), 2785 (w), 2174 (w), 1912 (w), 1658 (s), 1585 (m), 1493 (s), 1396 (s), 1301 (m), 1236 (s), 1154 (s), 1093 (s), 934 (sh), 902 (m), 829 (m), 662 (m), 624 (sh), 512 (w) (Figure S34).

#### 2.4.5 Synthesis of [(NH_2_)_3_C]_4_[{(4-F_3_C–C_6_H_4_)As}_2_Mo_6_O_24_] (**Gua-2**)

This polyanion was synthesized using the same procedure as that for **1**, but by using 0.027 g (0.10 mmol) of (4-trifluoromethylphenyl)arsonic acid (H_2_**L**_**CF3**_) instead of H_2_**L**_**F**_. Colorless crystals of **Gua-2** were observed the next day. Yield: 60 mg (73% based on Mo). Polyanion **2** is obtained in 90% reaction yield at room temperature, but heating to 80 °C resulted in 100% reaction yield. Elemental analysis calculated for **Gua-2**: Mo, 35.1; As, 9.14; F, 7.0; C, 13.18; H, 1.97; N, 10.25. Found: Mo, 35.5; As, 8.38; F, 7.3; C, 12.36; H, 2.43; N, 10.25. ^1^H NMR (D_2_O/H_2_O, 400 MHz, ppm): δ 7.83 (d, 2H, Ar), 8.31 (d, 2H, Ar) (Figure S11). ^19^F NMR (D_2_O/H_2_O, 376.5 MHz, ppm): δ –62.8 (Figure 2, red). FT-IR (2% KBr disk, cm^–1^): 3410 (br), 3364 (sh), 3256 (br), 3177 (br), 1661 (s), 1603 (sh),1568 (m), 1396 (s), 1324 (s), 1178 (s), 1127 (s), 1105 (sh), 1063 (s), 1023 (s), 944 (s), 913 (sh),895 (s), 836 (s), 792 (s), 650 (s), 530 (s), 466 (sh) (Figure S35).

#### 2.4.6 Synthesis of [(NH_2_)_3_C]_4_[{(4-F_3_CO–C_6_H_4_)As}_2_Mo_6_O_24_(H_2_O)]·4H_2_O (**Gua-3**)

This polyanion was synthesized using the same procedure as that for **1**, but by using 0.029 g (0.10 mmol) of (4-trifluoromethoxyphenyl)arsonic acid (H_2_**L**_**OCF3**_) instead of H_2_**L**_**F**_. Colorless crystals of **Gua-3** were observed the next day. Yield: 58 mg (66% based on Mo). Polyanion **3** is obtained in 100% reaction yield at room temperature, but the isolated yield of the guanidinium salt is higher when the reaction solution is heated at 80 °C. We do not know the reason for this, but we decided to report the latter condition in the experimental procedure above. Elemental analysis calculated for **Gua-3**: Mo, 32.7; As, 8.50; F, 6.47; C, 12.27; H, 2.40; N, 9.5. Found: Mo, 33.4; As, 8.08; F, 6.11; C, 12.84; H, 2.31; N, 9.4. ^1^H NMR (D_2_O/H_2_O, 400 MHz, ppm): δ 7.42 (d, 2H, Ar), 8.22 (d, 2H, Ar) (Figure S12). ^19^F NMR (D_2_O/H_2_O, 376.5 MHz, ppm): δ –57.4 (Figure 2, blue). FT-IR (2% KBr disk, cm^–1^): 3429 (br), 3343 (br), 3259 (sh), 3177 (br), 2790 (sh),1659 (s), 1579 (sh), 1500 (s), 1404 (m), 1297 (sh),1263 (s), 1212 (s), 1174 (s), 1095 (s), 1018 (m), 937 (sh),896 (s), 839 (s), 665 (s), 537 (sh), 501 (m) (Figure S36).

#### 2.4.7 Synthesis of Na_4_[{(4-F–C_6_H_4_)As}_4_Mo_12_O_46_]·15H_2_O (**Na-4**)

0.022 g (0.10 mmol) of (4-fluorophenyl)arsonic acid (H_2_**L**_**F**_) and 0.073 g (0.30 mmol) of Na_2_MoO_4_·2H_2_O were mixed in 5 mL of H_2_O and stirred at room temperature for a few minutes. After complete dissolution, the pH of the solution was adjusted to 1 with 2 M H_2_SO_4aq_. The solution was stirred at ∼80 °C for 1 h and then allowed to cool down to room temperature, filtered and kept for crystallization. Colorless crystals of **Na-4** were obtained after 10 days. Yield: 51 mg (70% based on Mo). Elemental analysis calculated for **Na-4**: Na, 3.14; Mo, 39.3; As, 10.2; F, 2.59; C, 9.84; H, 1.58. Found: Na, 3.25; Mo, 39.6; As, 10.2; F, 2.52; C, 10.03; H, 1.63. ^1^H NMR (D_2_O/H_2_O, 400 MHz, ppm): δ 7.26 (m, 2H, Ar), 8.85 (m, 2H, Ar) (Figure S13). ^19^F NMR (D_2_O/H_2_O, 376.5 MHz, ppm): δ –103.6 (Figure 4, black). FT-IR (2% KBr disk, cm^–1^): 3483 (br), 1624 (s), 1590 (s), 1497 (s), 1464 (sh),1402 (s), 1307 (s), 1241 (s), 1165 (s), 1085 (s), 981 (sh), 947 (w), 889 (s), 846 (s), 818 (sh), 702 (sh), 588 (s), 514 (m), 486 (s) (Figure S37).

#### 2.4.8 Synthesis of Na_2_(H_3_O)_2_[{(4-F_3_C–C_6_H_4_)As}_4_Mo_12_O_46_]·25H_2_O (**Na-5**)

This polyanion was synthesized using the same procedure as that for **4**, but by using 0.027 g (0.10 mmol) of (4-trifluoromethylphenyl)arsonic acid (H_2_**L**_**CF3**_), instead of H_2_**L**_**F**_. Colorless crystals of **Na-5** were obtained after 10 days. Yield: 28 mg (34% based on Mo). Elemental analysis calculated for **Na-5**: Na, 1.39; Mo, 34.9; As, 9.08; F, 6.9; C, 10.19; H, 2.20. Found: Na, 1.77; Mo, 34.6; As, 9.25; F, 7.6; C, 10.25; H, 2.06. ^1^H NMR (D_2_O/H_2_O, 400 MHz, ppm): δ 7.85 (d, 2H, Ar), 8.99 (d, 2H, Ar) (Figure S14). ^19^F NMR (D_2_O/H_2_O, 376.5 MHz, ppm): δ –63.2 (Figure 4, red). FT-IR (2% KBr disk, cm^–1^): 3479 (br), 1626 (s), 1405 (s), 1327 (s), 1180 (s), 1133 (s), 1061 (s), 1020 (s), 983 (sh), 956 (s), 927 (s), 878 (s), 838 (m), 696 (s), 630 (sh), 592 (s), 502 (sh), 484 (s), 441 (s) (Figure S38).

#### 2.4.9 Synthesis of Na_2_(H_3_O)_2_[{(4-F_3_CO–C_6_H_4_)As}_4_Mo_12_O_46_]·17H_2_O (**Na-6**)

This polyanion was synthesized using the same procedure as that for **4**, but by using 0.029 g (0.10 mmol) of (4-trifluoromethoxyphenyl)arsonic acid (H_2_**L**_**OCF3**_) instead of H_2_**L**_**F**_. Colorless crystals of **Na-6** were obtained after 10 days. Yield: 29 mg (36% based on Mo). Elemental analysis calculated for **Na-6**: Na, 1.43; Mo, 35.74; As, 9.3; F, 7.08; C, 10.44; H, 1.75. Found: Na, 1.89; Mo, 34.9; As, 9.84; F, 7.15; C, 11.8; H, 1.35. ^1^H NMR (D_2_O/H_2_O, 400 MHz, ppm): δ 7.42 (d, 2H, Ar), 8.88 (d, 2H, Ar) (Figure S15). ^19^F NMR (D_2_O/H_2_O, 376.5 MHz, ppm): δ –57.4 (Figure 4, blue). FT-IR (2% KBr disk, cm^–1^): 3471.8 (br), 3102 (w), 1628 (s), 1594 (sh), 1500 (s), 1411 (w), 1383 (sh), 1324 (m), 1291 (m), 1264 (s), 1212 (s), 1171 (m), 1089 (m), 1063 (sh), 1019 (w), 980 (m), 952 (s), 928 (s), 867 (s), 840 (sh), 668 (m), 590 (s), 511 (sh), 488 (s), 419 (m) (Figure S39).

## 3 Results and Discussion

### 3.1 Structure and Characterization of Polyanions **1–3**

**Figure 1:**
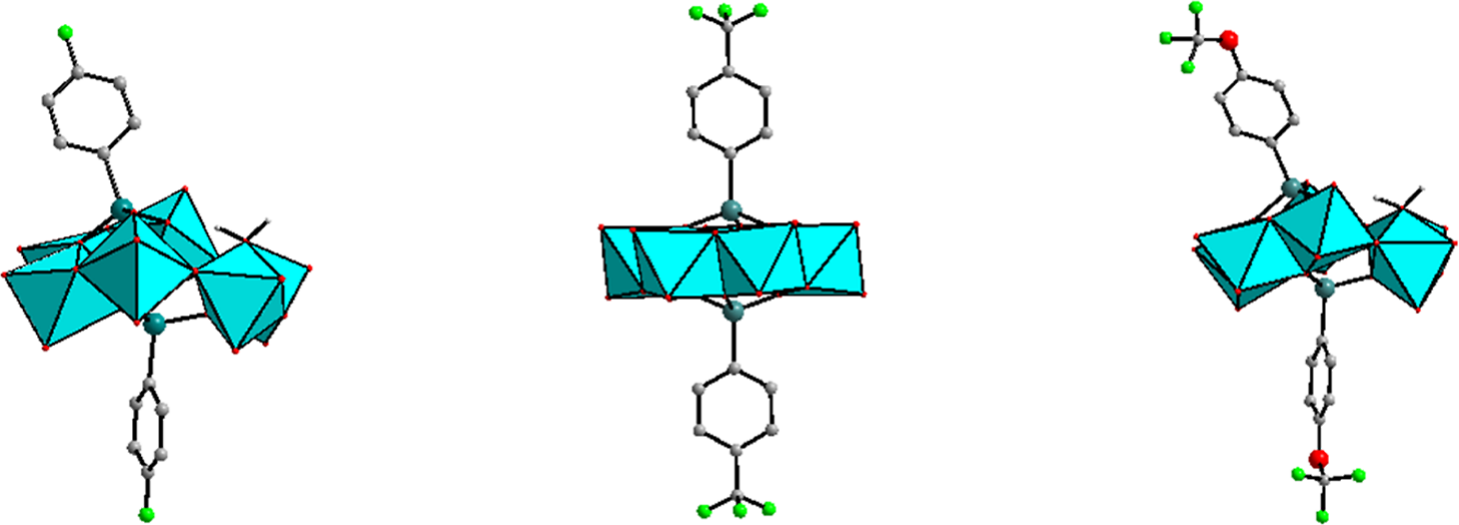
Structures of polyanions **1** (left), **2** (middle) and **3** (right). Color code: MoO_6_ octahedra, cyan; As, teal; O, red; F, green; C, gray and H, white. Hydrogen atoms are omitted for clarity.

The polyanion [{(4-F–C_6_H_4_)As}_2_Mo_6_O_24_(H_2_O)]^4–^ (**1**) was synthesized by reacting (4-fluorophenyl)arsonic acid (H_2_**L**_**F**_) with sodium molybdate in aqueous solution at pH 2.5 to 3.5 with heating at ∼80 °C for 1 h, and then isolated as the hydrated guanidinium salt [(NH_2_)_3_C]_4_[{(4-F–C_6_H_4_)As}_2_Mo_6_O_24_(H_2_O)]·4H_2_O (**Gua-1**), which crystallized in the monoclinic system with space group *Cc* (Table 1). Polyanion **1** is composed of six MoO_6_ octahedra connected by corner-, edge-, and face-sharing forming a bent ring, which is capped on each side by an arsonate hetero group **L**_**F**_^2–^ (Figure 1, left). However, the bonding pattern of the two arsonate groups to the molybdenum-oxo core is different, on one side of the ring the arsonate group is connected to all six Mo centers and on the other side the arsonate group is connected to only four Mo centers, resulting in a bent ring. The face-sharing of two adjacent MoO_6_ units in **1** is facilitated by a bridging water molecule (O_23_) [bond length 2.407(8)–2.436(8) Å]. This polyanion comprises terminal oxo ligands with Mo–O bond lengths from 1.696(8) to 1.743(8) Å, doubly bridging μ_2_-oxo ligands with Mo–O(Mo) bond lengths of 1.889(9)–1.986(8) Å, doubly bridging μ_2_-oxo ligands with Mo–O(As) bond lengths of 2.181(8) to 2.222(9) Å and triply bridging μ_3_-oxo ligands with Mo–O(As) bond lengths from 2.239(8) to 2.366(8) Å. The arsenic atoms adopt a tetrahedral geometry with As–O bond lengths from 1.675(9) to 1.705(8) Å, along with As–C bond lengths of 1.883(12)–1.912(12) Å and O–A–O bond angles between 107.1(4) and 113.5(4)°. In the solid state, each polyanion **1** was found to be surrounded by four guanidinium counter cations, along with some disordered lattice water molecules. The XRD data confirmed that the solid-state structure of **Gua-1** is stabilized by hydrogen bonding interactions between the guanidinium cations and the oxygen atoms of polyanion **1**. The precise formula unit of the salt **Gua-1** was determined by elemental analysis and TGA on bulk material (Figure S40). The TGA of **Gua-1** shows a 5.1% weight loss until 150 °C, corresponding to a loss of 5 water molecules, which means 4 lattice water molecules and one coordinated water molecule (as seen in the crystal structure). Then the compound remains stable up to 300 °C, after which it starts to decompose.

The polyanion [{(4-F_3_C–C_6_H_4_)As}_2_Mo_6_O_24_]^4–^ (**2**) was synthesized by reacting (4-trifluoromethylphenyl)arsonic acid (H_2_**L**_**CF3**_) with sodium molybdate in an aqueous solution using conditions as for polyanion **1**. Polyanion **2** crystallized in the monoclinic system, space group *P*2_1_/*n* and was isolated as a guanidinium salt, [(NH_2_)_3_C]_4_[{(4-F_3_C–C_6_H_4_)As}_2_Mo_6_O_24_] (**Gua-2**), see Table 1. Unlike **1**, polyanion **2** comprises a flat ring of six edge-shared MoO_6_ octahedra, which is capped on each side by a **L**_**CF3**_^2–^ hetero group (Figure 1, middle). The Mo–O, As–O and As–C bond lengths in **2** are in the usual ranges (see cif file for details). In analogy to **1**, the solid-state structure of **2** exhibits four guanidinium cations around each polyanion. The precise formula unit of the salt **Gua-2** was determined by elemental analysis and TGA on bulk material (Figure S41). The TGA shows no weight loss until 300 °C, which confirms the absence of lattice water and then the degradation of the POM structure starts.

The polyanion [{(4-F_3_CO–C_6_H_4_)As}_2_Mo_6_O_24_(H_2_O)]^4–^ (**3**) was prepared by reaction of (4-trifluoromethoxyphenyl)arsonic acid (H_2_**L**_**OCF3**_) and sodium molybdate using the same conditions as for **1**. In fact, polyanion **3** is isostructural with **1**, the only difference being the *para* functionality on the phenyl ring, which is F for **1** and OCF_3_ for **3** (Figure 1, right). Hence, the As–O/As–C and Mo–O bond lengths in **3** and **1** are very similar. Moreover, the guanidinium salt of this polyanion, [(NH_2_)_3_C]_4_[{(4-F_3_CO–C_6_H_4_)As}_2_Mo_6_O_24_(H_2_O)]·4H_2_O (**Gua-3**), is isomorphous with **Gua-1** (see Figure S42 for the thermogram). The thermogram displays an initial weight loss of ∼4.5% until 150 °C, indicating the loss of 5 water molecules. Then the structure is stable up to 290 °C and after that starts to collapse.

**Figure 2:**
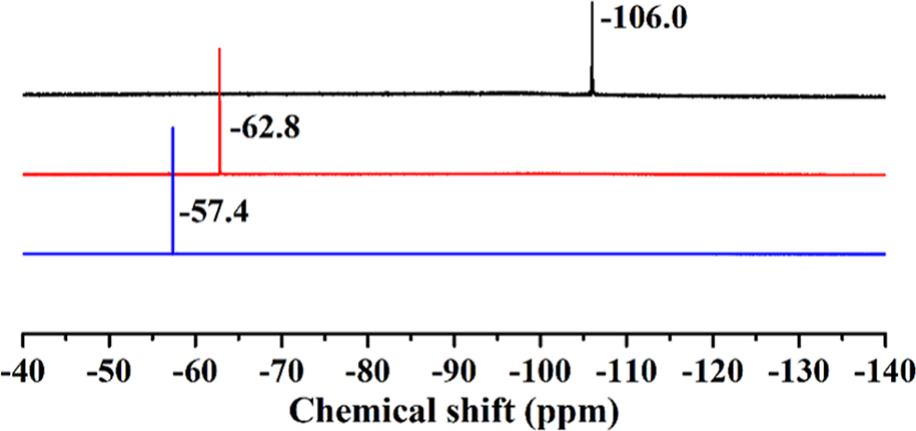
^19^F NMR spectra of polyanions **1** (black), **2** (red), and **3** (blue), after dissolving the respective guanidinium salts in water.

All three polyanions **1**–**3** are (sparingly) soluble and solution-stable in water and hence we performed detailed multinuclear (^1^H, ^19^F) NMR studies. The ^19^F NMR spectra for **1**–**3** are shown in Figure 2, indicating the expected resonances at: δ –106.0 ppm for **1**, –62.8 ppm for **2**, and –57.4 ppm for **3** (see also Figures S10–S12 for the ^1^H spectra of polyanions **1**–**3**). We compare the ^1^H and ^19^F NMR spectra of polyanions **1**–**3** with the free capping groups at the respective pH values (see Figures S16–S21). Then, we decided to determine the reaction yield in solution by monitoring the ^1^H and ^19^F NMR spectra of fresh reaction solutions for all three polyanions after adjustment to pH 3 at three different temperatures: room temperature, 50 and 80 °C, at different time intervals. For polyanions **1** and **3**, the NMR data indicated essentially 100% reaction yield by simple pH adjustment at room temperature, suggesting an instantaneous self-assembly process of the reagents. For polyanion **2**, the NMR data showed around 90% yield about 30 min after pH adjustment at room temperature (see Figures S22–S27). We conclude that all three polyanions **1**–**3** can be formed easily in a pure form and high yield by simple mixing of sodium molybdate with the organoarsonic acids, at pH 3 at room temperature, which is rare in POM chemistry.

### 3.2 Structure and Characterization of Polyanions **4–6**

We discovered that a slight pH variation from 3 to 1 during the synthesis of **1** allowed us to prepare a larger polyanion. In fact, the 12-molybdate [{(4-F–C_6_H_4_)As}_4_Mo_12_O_46_]^4–^ (**4**) was synthesized by reacting (4-fluorophenyl)arsonic acid (H_2_**L**_**F**_) with sodium molybdate in aqueous solution at pH 1 at ∼80 °C after 1 h.

**Figure 3:**
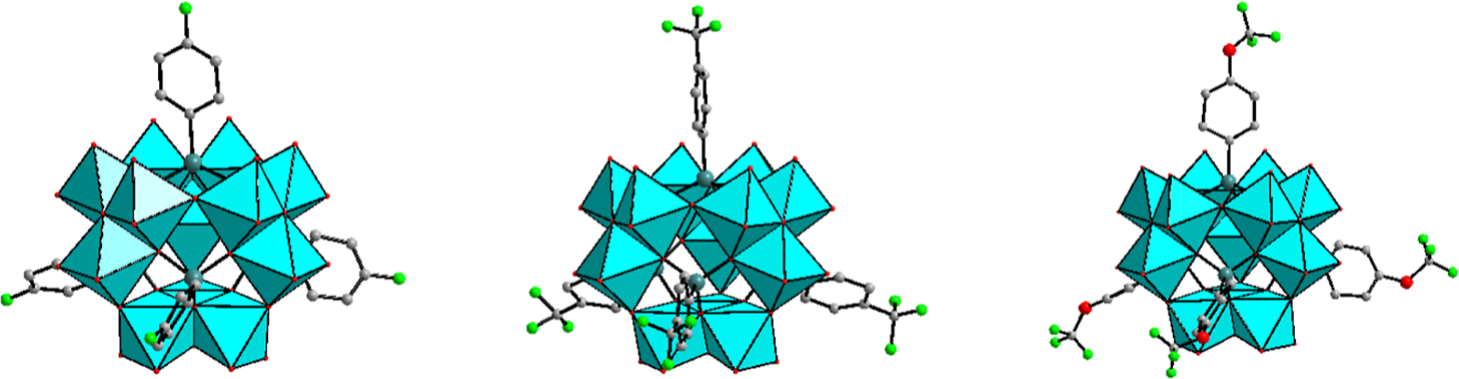
Structures of polyanions **4** (left), **5** (middle) and **6** (right). Color code: MoO_6_ octahedra, cyan; As, teal; O, red; F, green and C, gray. Hydrogen atoms are omitted for clarity.

After the reaction, the resulting polyanion **4** was isolated as a sodium salt, Na_4_[{(4–F–C_6_H_4_)As}_4_Mo_12_O_46_]·15H_2_O (**Na-4**), with a monoclinic crystal system and space group *C*2/*c* (Table 1). The inverted-Keggin polyanion **4** contains four edge-shared Mo_3_O_13_ units, interconnected via corners and all four faces lined by three such units are capped by an arsonate group each (Figure 3, left). This polyanion exhibits terminal oxygen atoms with Mo–O bond lengths in the range of 1.669(8) to 1.709(9) Å, doubly bridging oxygen atoms with Mo–O(Mo) bond lengths ranging from 1.860(7) to 1.886(8) Å, triply bridging oxygen atoms with Mo–O(Mo) bond lengths ranging from 2.022(8) to 2.059(7) Å and triply bridging oxygen atoms with Mo–O(As) bond lengths ranging from 2.263(7) to 2.318(7) Å. The arsenic atoms adopt a tetrahedral geometry with As–O bond lengths ranging from 1.666(7) to 1.701(6) Å, along with As–C bond lengths of 1.866(12) to 1.916(13) Å and O–As–O bond angles between 107.1(4) and 110.9(3)°. In the solid state, each polyanion **4** is surrounded by four sodium counter cations, along with some disordered lattice water molecules. The precise formula unit Na_4_[{(4–F–C_6_H_4_)As}_4_Mo_12_O_46_]·15H_2_O (**Na-4**) was determined by elemental analysis.

For the other two fluorinated arsonate derivatives we could also prepare the larger 12-molybdates at pH 1, otherwise keeping the reaction conditions identical to **4**. This reemphasizes the importance of pH in POM synthesis. The 12-molybdate polyanion [{(4-F_3_C–C_6_H_4_)As}_4_Mo_12_O_46_]^4–^ (**5**) was synthesized by reacting (4-trifluoromethylphenyl)arsonic acid (H_2_**L**_**CF3**_) with sodium molybdate in an aqueous solution using conditions as for polyanion **4**. The structure of polyanion **5** is identical to that of **4**, the only difference being the *para* functionality on the phenyl ring, which is F for **4** and CF_3_ for **5** (Figure 3, left and middle). The 12-molybdate polyanion [{(4-F_3_CO–C_6_H_4_)As}_4_Mo_12_O_46_]^4–^ (**6**) was prepared by reacting (4-trifluoromethoxyphenyl)arsonic acid (H_2_**L**_**OCF3**_) with sodium molybdate in an aqueous solution using the same conditions as for **4**. The structure of polyanion **6** is also identical to that of **4** and **5**, but the *para* functionality on the phenyl ring is OCF_3_ (Figure 3, right). The As–O/As–C and Mo–O bond lengths in **4**–**6** are very similar and the sodium salts **Na-4**, **Na-5**, and **Na-6** are isomorphous. The thermograms of the three compounds are shown in Figures S43–S45. The thermogram of **Na-4** shows ∼9.5% weight loss corresponding to 15 lattice water molecules and at 350 °C the structure starts to degrade. The TGA of **Na-5** displays ∼14.5% weight loss until 150 °C corresponding to the loss of 27 water molecules and at 300 °C the POM structure starts to degrade. For **Na-6**, the thermogram shows ∼10.4% weight loss corresponding to the loss of 19 water molecules and at 260 °C the structure starts to collapse.

**Figure 4:**
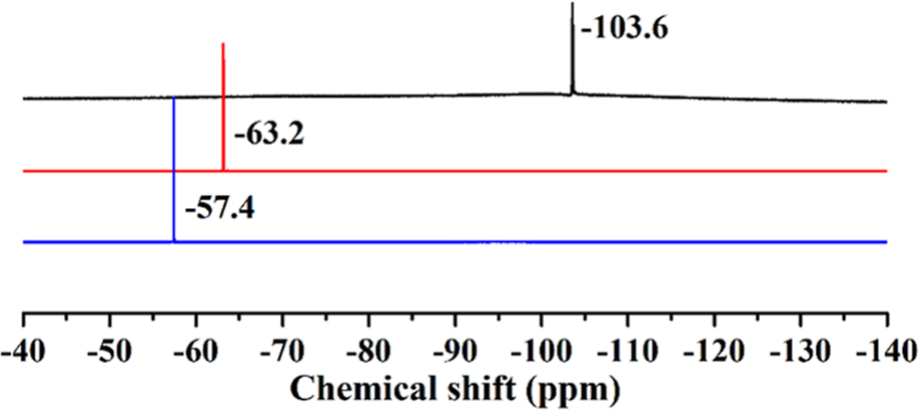
^19^F NMR spectra of polyanions **4** (black), **5** (red), and **6** (blue), after dissolving the respective sodium salts in water.

The salts of all three polyanions **4**–**6** are soluble in water and hence we performed multinuclear (^1^H, ^19^F) NMR studies. The ^19^F NMR spectra for **4**–**6** are shown in Figure 4, indicating the expected resonances at δ –103.6 ppm for **4**, –63.2 ppm for **5** and –57.4 ppm for **6** (see also Figures S13–S15 for the ^1^H spectra of polyanions **4**–**6**). We compared the ^1^H and ^19^F NMR spectra of polyanions **4**–**6** with the free capping groups at the respective pH (see Figures S16–S21). Then, we estimated the reaction yield in solution by monitoring the ^1^H and ^19^F NMR spectra of fresh reaction solutions for all three polyanions after adjustment to pH 1 at three different temperatures (room temperature, 50 and 80 °C) and at different time intervals. For polyanion **4**, the NMR data indicated almost 100% yield after 1 h heating at 80 °C, whereas for polyanion **5** we noticed a 85% yield after 1 h heating at 80 °C, and finally, for polyanion **6**, the yield was almost 100% after 30 min heating at 80 °C (see Figures S28–S33). This observation indicates that also polyanions **4**–**6** can be formed easily and in high yield in solution via a self-assembly process.

**Figure 5:**
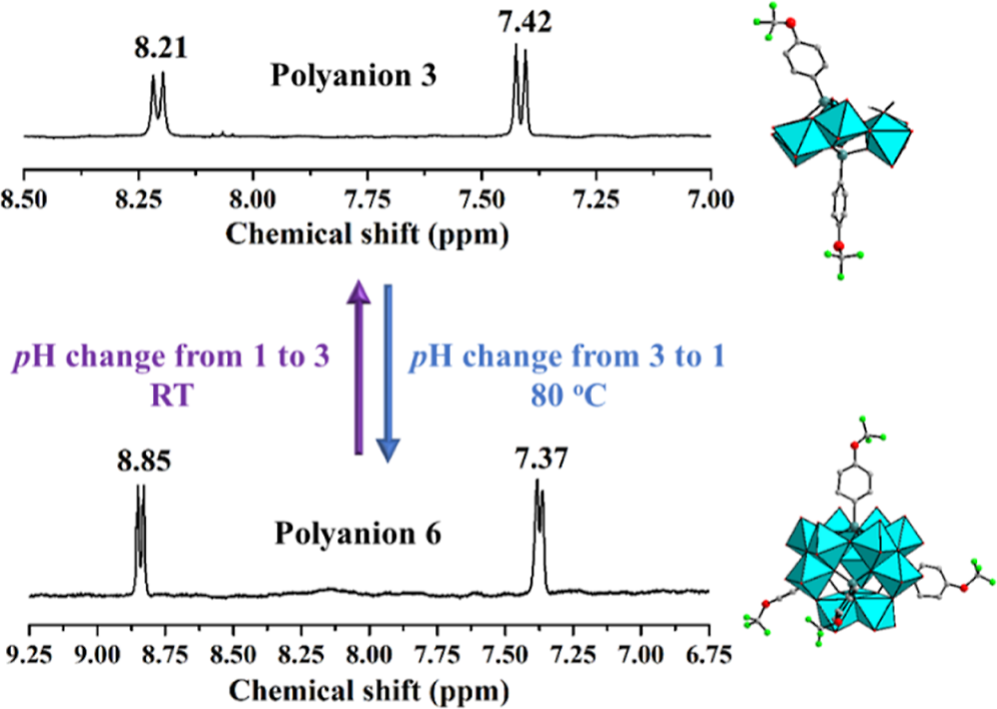
Solution ^1^H NMR spectra of freshly synthesized polyanions **3** and **6** and the pH/T conditions to easily interconvert **6** to **3** and **3** to **6** reversibly in solution.

In summary, we have prepared the 6-molybdates **1**–**3** and the 12-molybdates **4**–**6** by simple reaction of the respective fluorinated phenylarsonate with sodium molybdate in water. The 6-molybdates are formed at pH 3 whereas the 12-molybdates are formed in more acidic medium at pH 1. Both structure types have the exact same ratio of hetero groups to addenda (6:2 or 12:4). Hence the interconversion of the 6-molybdate series **1**–**3** to the corresponding 12-molybdate series **4**–**6** by changing the pH from 3 to 1 occurs quantitatively, without any side products being formed. Furthermore, such transformation is reversible. When considering experimental details, polyanion **3** is fully transformed to polyanion **6** upon changing the pH from 3 to 1 after 30 min heating at 80 °C. An increase of the pH of such solution from 1 to 3 instantly converts polyanion **6** to polyanion **3** at room temperature (Figure 5). In general, the conversion of the 12-molybdates **4**–**6** to the 6-molybdates **1**–**3** is easier than the other way around. We also tested the antibacterial activity of all six polyanions **1**–**6** for *Escherichia coli* (Gram-negative) and *Bacillus subtilis* (Gram-positive), but no significant activity was detected.

## 4 Conclusions

We have successfully synthesized and characterized two new arylarsonic acids, (4-F_3_C–C_6_H_4_)AsO_3_H_2_ (H_2_**L**_**CF3**_) and (4-F_3_CO–C_6_H_4_)AsO_3_H_2_ (H_2_**L**_**OCF3**_). By reacting these as well as the previously reported (4-F–C_6_H_4_)AsO_3_H_2_ (H_2_**L**_**F**_) with sodium molybdate in acidic aqueous solution, six new polyoxomolybdates were synthesized, the 6-molybdates [{(4-F–C_6_H_4_)As}_2_Mo_6_O_24_(H_2_O)]^4–^ (**1**), [{(4-F_3_C–C_6_H_4_)As}_2_Mo_6_O_24_]^4–^ (**2**) and [{(4-F_3_CO–C_6_H_4_)As}_2_Mo_6_O_24_(H_2_O)]^4–^ (**3**) as well as the 12-molydates [{(4-F–C_6_H_4_)As}_4_Mo_12_O_46_]^4–^ (**4**), [{(4-F_3_C–C_6_H_4_)As}_4_Mo_12_O_46_]^4–^ (**5**) and [{(4-F_3_CO–C_6_H_4_)As}_4_Mo_12_O_46_]^4–^ (**6**). Solution NMR studies during the reaction process indicated that the 6-molybdates **1**–**3** are formed quickly (essentially just after pH adjustment) in around 100% yield at room temperature, whereas the 12-molybdates are formed in around 90–95% yield within an hour at 80 °C. The Mo_6_ polyanions **1**–**3** and the Mo_12_ polyanions **4**–**6** can be interconverted reversibly as a function of pH (3 vs 1). It is worth noting that all six polyanions possess fluorinated functional groups (F, F_3_C-, F_3_CO−) in the *para* position of the phenyl ring, which carry the potential for interaction with biomolecules such as peptides, nucleotides, antibodies, or nanoparticles. This opens exciting possibilities for polyanions **1**–**6** in various biomedical applications, which are planned for the future. Currently, we are in the process of preparing other fluorinated polyoxometalates and investigating their properties.

## Supporting Information Available

NMR and FT-IR spectra, thermograms (PDF)
