# Supplementary material for: Fluorinated Arylarsonate-Containing Polyoxomolybdates: pH-Dependent Formation of Mo6 vs Mo12 Species and Their Solution Properties
Source: Inorg Chem. 2024 Sep 26;63(40):18838–46. doi: 10.1021/acs.inorgchem.4c02951 (PMC11462504; doi:10.1021/acs.inorgchem.4c02951)
Supplement: Supplementary file 1 — ic4c02951_si_001.pdf [file ic4c02951_si_001.pdf]

Associated Content

Supporting Information

Fluorinated Arylarsonate-Containing Polyoxomolybdates: pH-dependent Formation of Mo_6 *vs* Mo_{12} Species and Their Solution Properties

Arun Pal,[a] Saurav Bhattacharya,[a,b] Xiang Ma,[a,c] Ahmad Ben Kiran,[d] Cristian Silvestru,[d] and Ulrich Kortz*[a]

[a] *School of Science, Constructor University, Campus Ring 1, 28759 Bremen, Germany,* Email: ukortz@constructor.university

[b] *Department of Chemistry, BITS Pilani K. K. Birla Goa Campus, 403726 Goa, India*

[c] *Fujian Provincial Key Laboratory of Advanced Inorganic Oxygenated Materials, College of Chemistry, Fuzhou University, Fuzhou 350108, Fujian, China*

[d] *Department of Chemistry, Supramolecular Organic and Organometallic Chemistry Centre (SOOMCC), Faculty of Chemistry and Chemical Engineering, Babeş-Bolyai University, 11 Arany Janos, 400028 Cluj-Napoca, Romania*

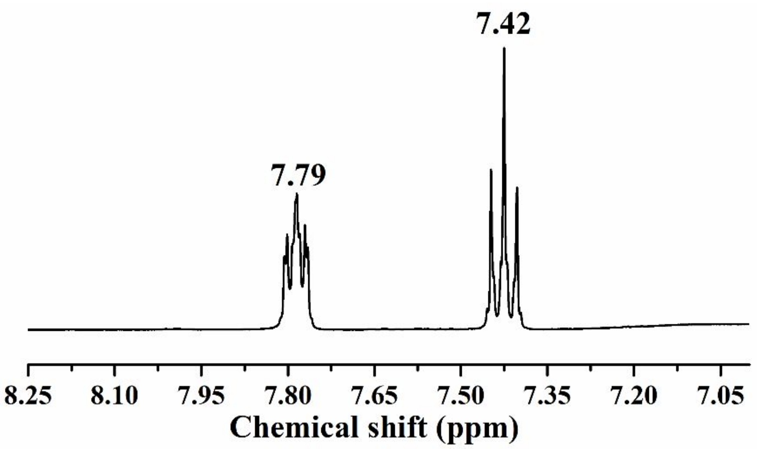

Figure S1. ^1H NMR of (4-fluorophenyl)arsonic acid, (4-F-C_6H_4)AsO_3H_2 (H_2**L_F**), recorded at room temperature in DMSO-d_6.

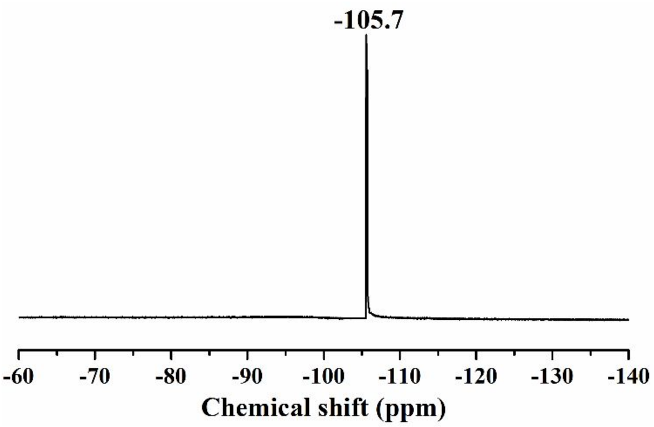

Figure S2. ^{19}F NMR of $H_2\mathbf{L_F}$, recorded at room temperature in DMSO-d_6.

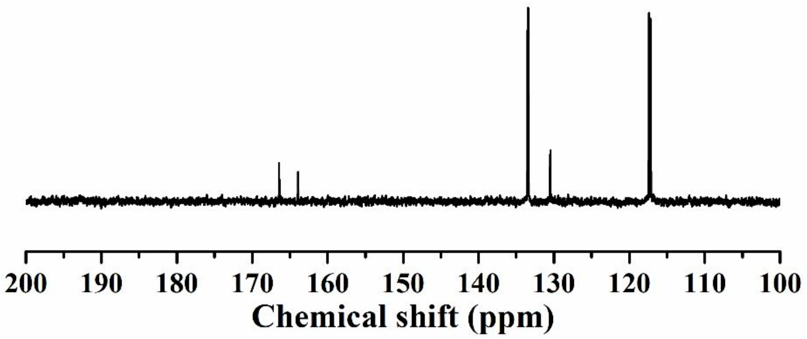

Figure S3. $^{13}C\{^1H\}$ NMR of $H_2\mathbf{L_F}$, recorded at room temperature in DMSO-d_6.

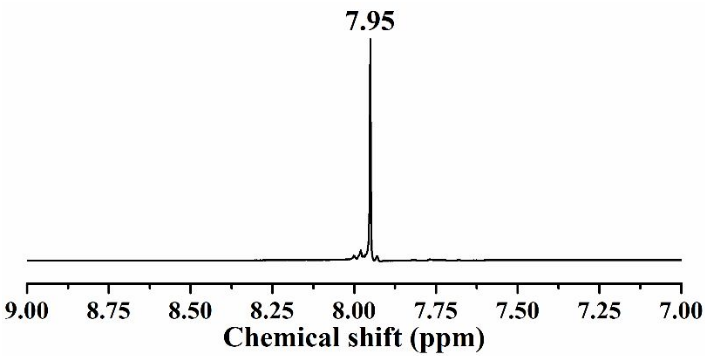

Figure S4. ^{1}H NMR of (4-trifluoromethylphenyl)arsonic acid, (4-F_3C-C_6H_4)AsO_3H_2 ($H_2\mathbf{L_{CF3}}$), recorded at room temperature in DMSO-d_6.

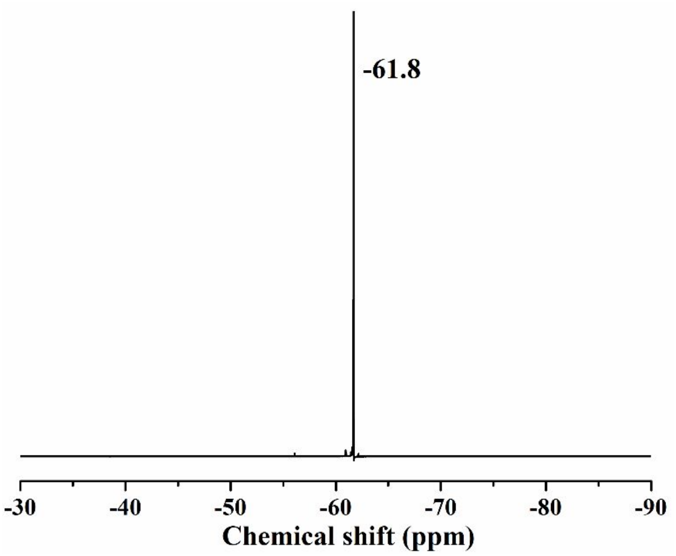

Figure S5. ^{19}F NMR of $H_2\mathbf{L}_{CF3}$, recorded at room temperature in DMSO-*d*$_6$.

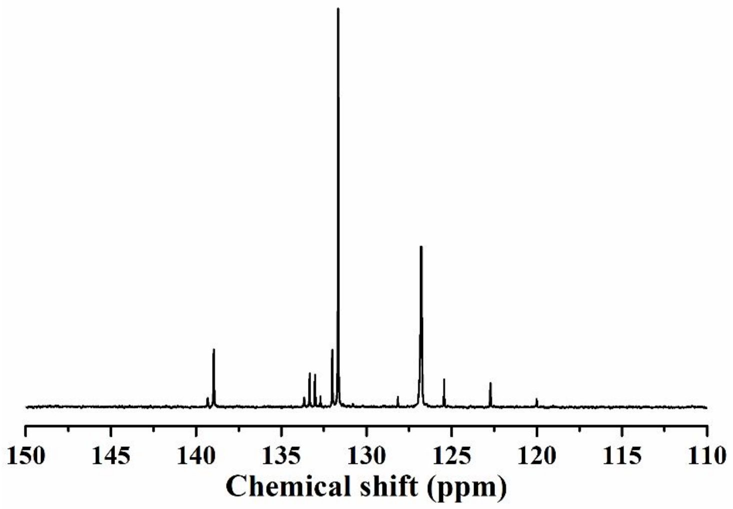

Figure S6. $^{13}C\{^1H\}$ NMR of $H_2\mathbf{L}_{CF3}$, recorded at room temperature in DMSO-*d*$_6$.

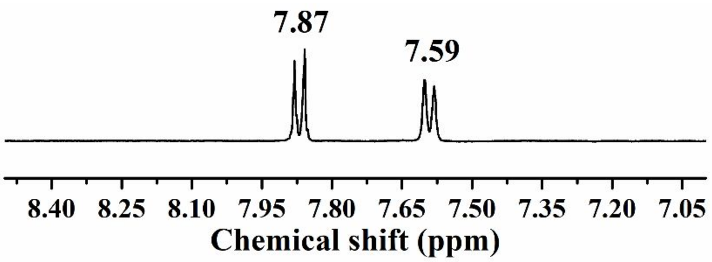

Figure S7. ^1H NMR of (4-trifluoromethoxyphenyl)arsonic acid, $(4\text{-}F_3CO\text{-}C_6H_4)AsO_3H_2$ ($H_2\mathbf{L}_{OCF3}$), recorded at room temperature in DMSO-*d*$_6$.

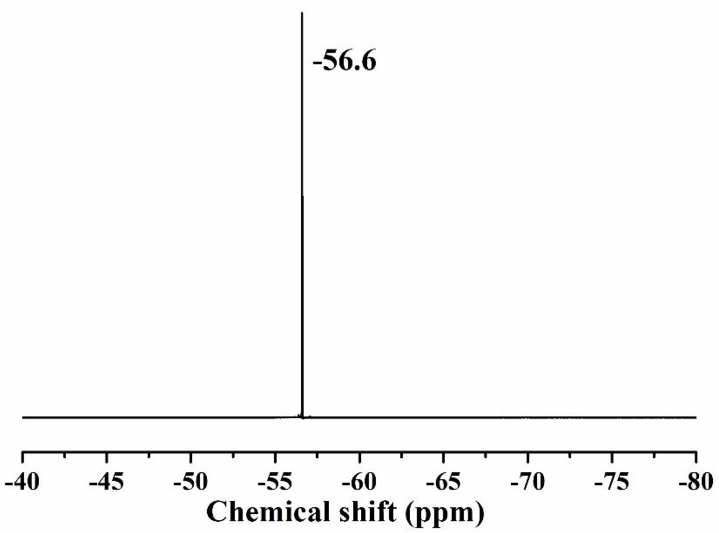

Figure S8. ^{19}F NMR of H_2**L$_{OCF3}$**, recorded at room temperature in DMSO-d_6.

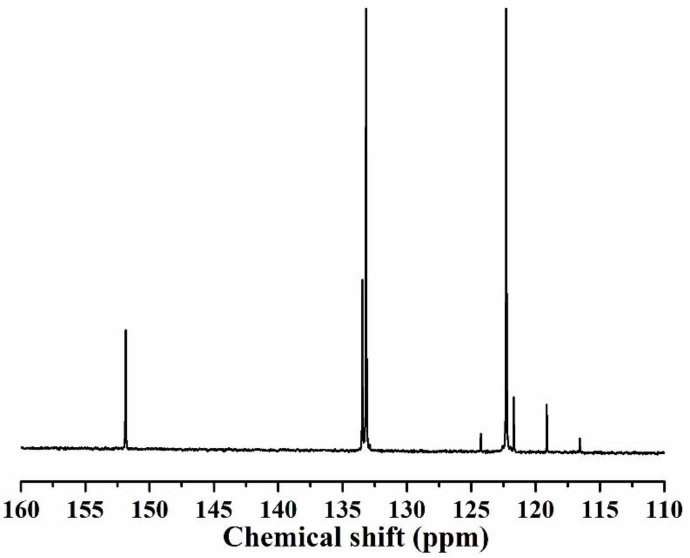

Figure S9. ^{13}C{^{1}H} NMR of H_2**L$_{OCF3}$**, recorded at room temperature in DMSO-d_6.

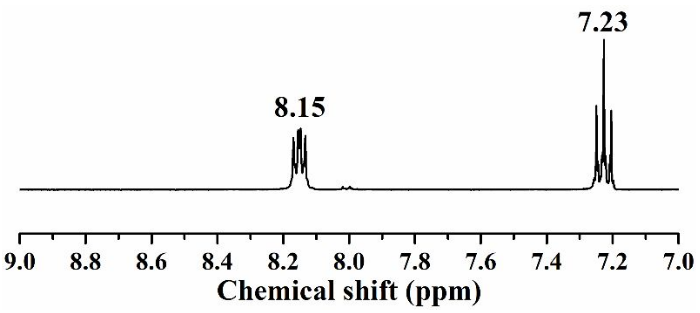

Figure S10. ^{1}H NMR of **Gua-1**, recorded at room temperature in H_2O-D_2O mixture.

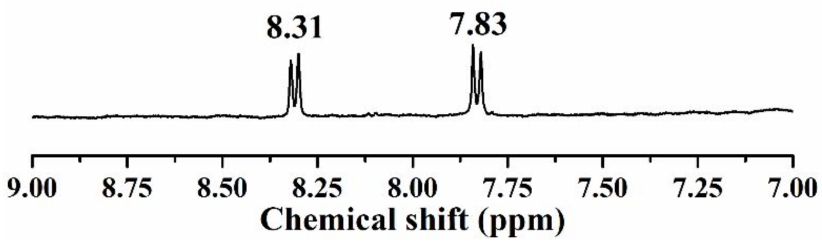

Figure S11. ^{1}H NMR of **Gua-2**, recorded at room temperature in H_2O-D_2O mixture.

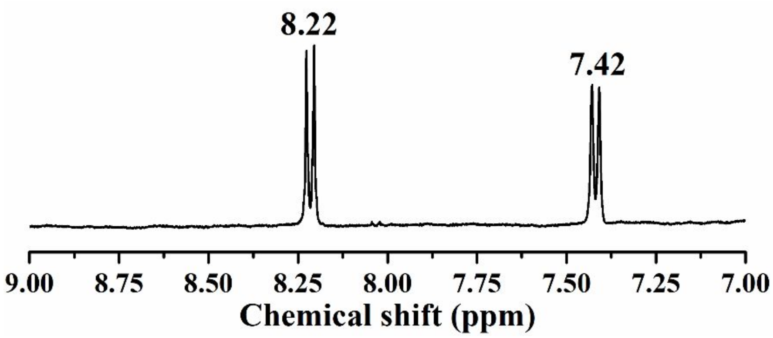

Figure S12. ^{1}H NMR of **Gua-3**, recorded at room temperature in H_2O-D_2O mixture.

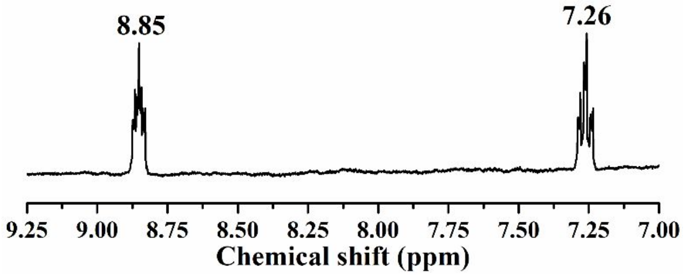

Figure S13. ^{1}H NMR of **Na-4**, recorded at room temperature in H_2O-D_2O mixture.

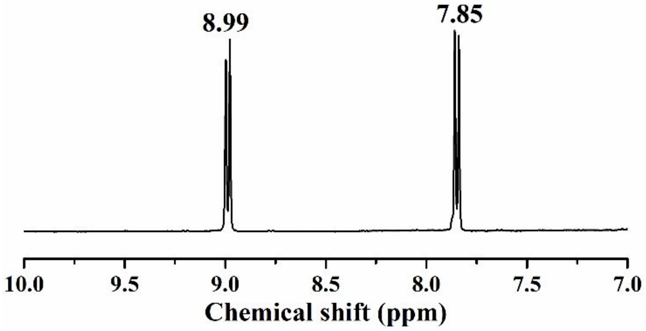

Figure S14. ^{1}H NMR of **Na-5**, recorded at room temperature in H_2O-D_2O mixture.

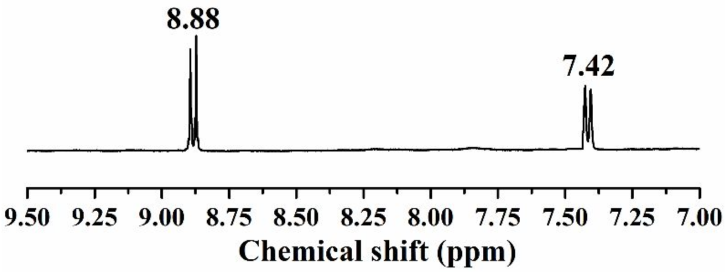

Figure S15. ^{1}H NMR of **Na-6**, recorded at room temperature in H_2O-D_2O mixture.

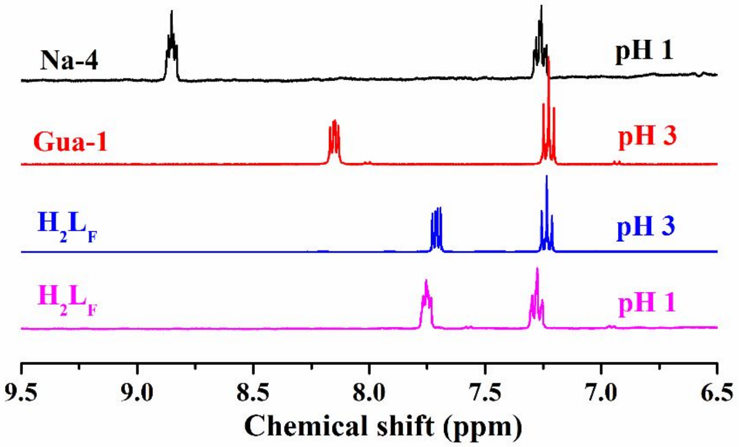

Figure S16. ^{1}H NMR spectra of **Gua-1**, **Na-4**, and H_2**L**$_F$ at their respective pH, recorded at room temperature in H_2O.

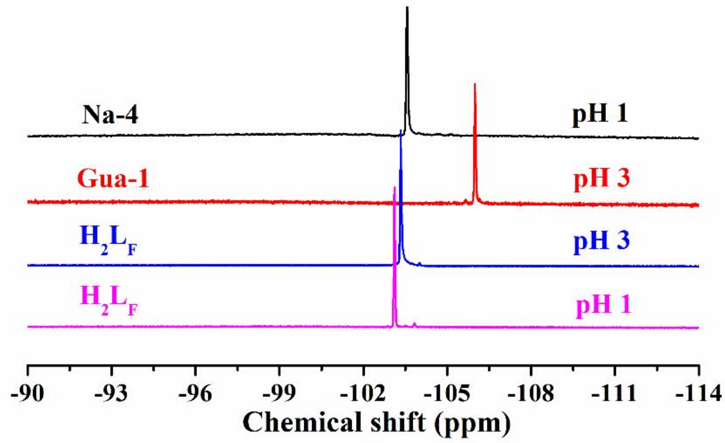

Figure S17. ^{19}F NMR spectra of **Gua-1**, **Na-4**, and H_2**L**$_F$ at their respective pH, recorded at room temperature in H_2O.

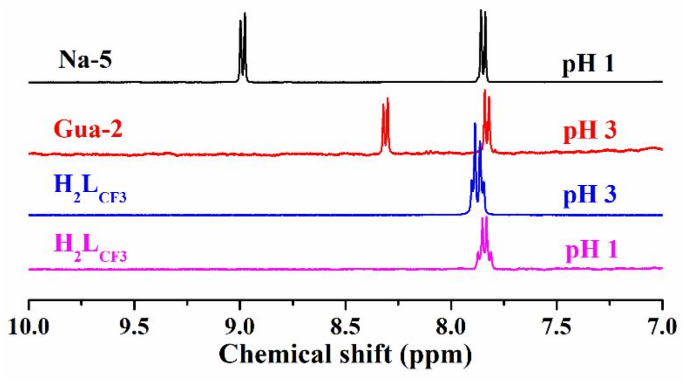

Figure S18. ^{1}H NMR spectra of **Gua-2**, **Na-5**, and H_2**L$_{CF3}$** at their respective pH, recorded at room temperature in H_2O.

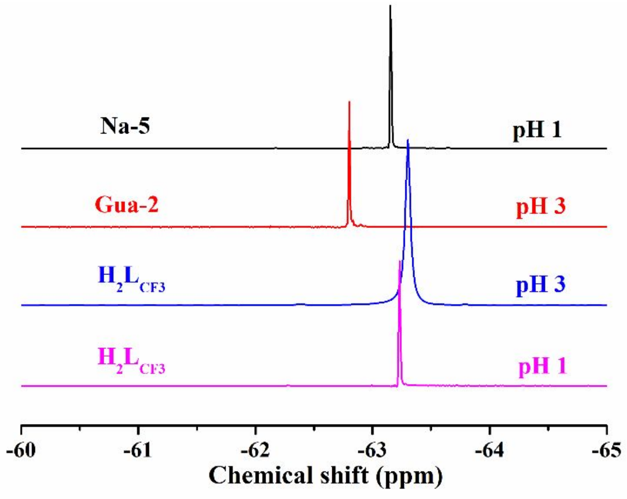

Figure S19. ^{19}F NMR spectra of **Gua-2**, **Na-5**, and H_2**L$_{CF3}$** at their respective pH, recorded at room temperature in H_2O.

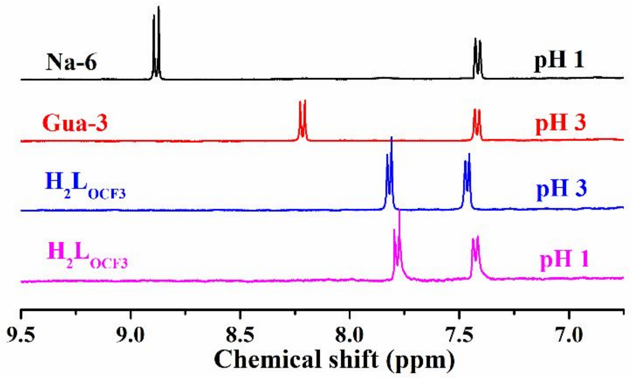

Figure S20. ^{1}H NMR spectra of **Gua-3**, **Na-6**, and H_2**L$_{OCF3}$** at their respective pH, recorded at room temperature in H_2O.

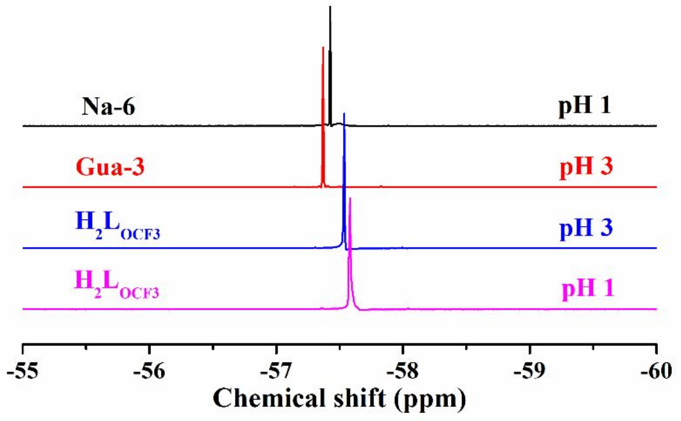

Figure S21. ^{19}F NMR spectra of **Gua-3**, **Na-6**, and H_2**L**$_{OCF3}$ at their respective pH, recorded at room temperature in H_2O.

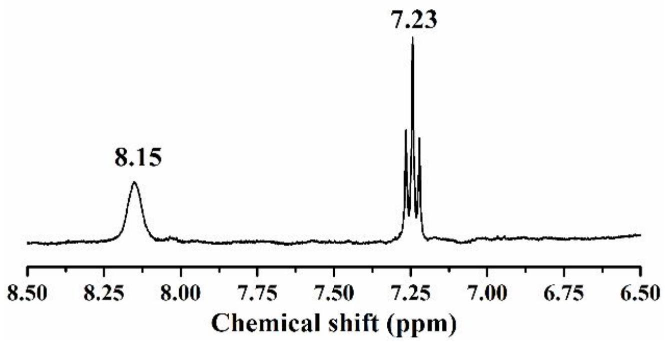

Figure S22. ^{1}H NMR of fresh reaction solution of **1** after adjustment of the required pH recorded at room temperature in H_2O-D_2O mixture.

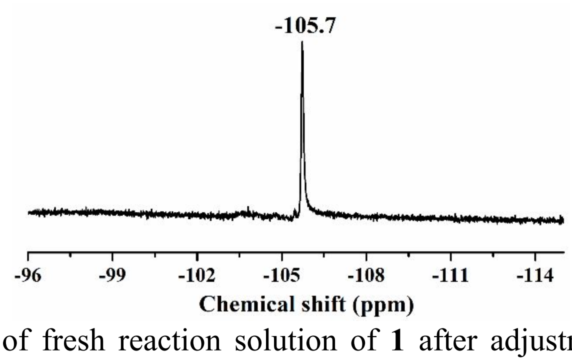

Figure S23. ^{19}F NMR of fresh reaction solution of **1** after adjustment of the required pH recorded at room temperature in H_2O-D_2O mixture.

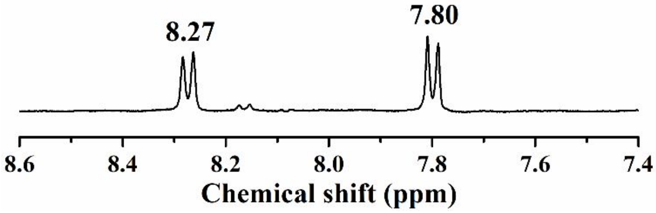

Figure S24. ^{1}H NMR of fresh reaction solution of **2** after 30 min of adjustment of the required pH recorded at room temperature in H_2O-D_2O mixture.

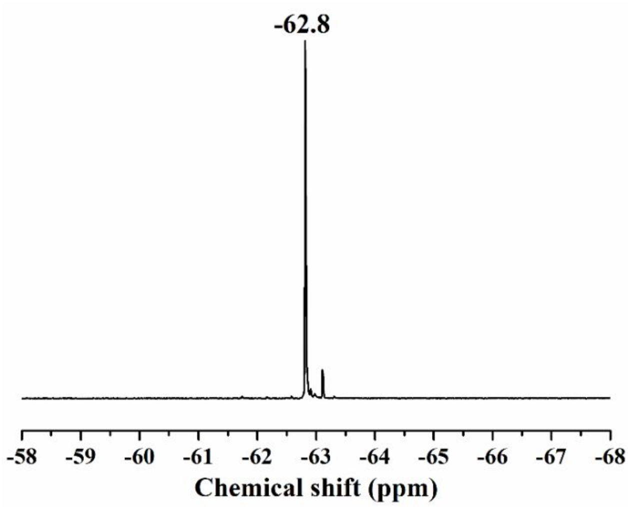

Figure S25. ^{19}F NMR of fresh reaction solution of **2** after 30 min of adjustment of the required pH recorded at room temperature in H_2O-D_2O mixture.

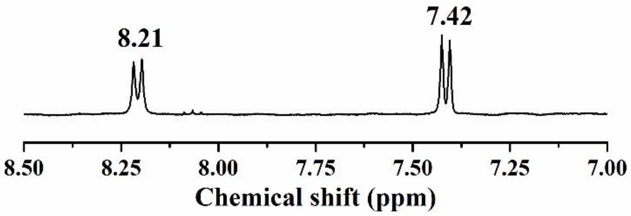

Figure S26. ^{1}H NMR of fresh reaction solution of **3** after adjustment of the required pH recorded at room temperature in H_2O-D_2O mixture.

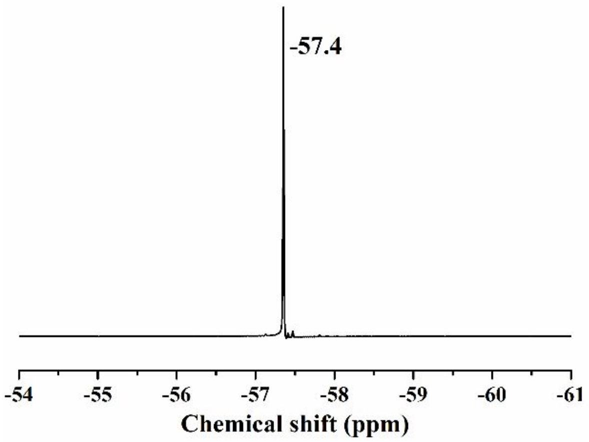

Figure S27. ^{19}F NMR of fresh reaction solution of **3** after adjustment of the required pH recorded at room temperature in H_2O-D_2O mixture.

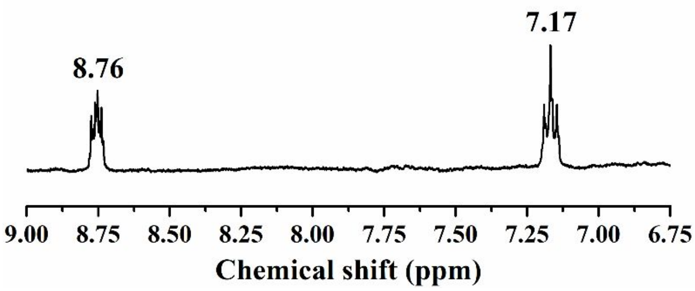

Figure S28. ^{1}H NMR of fresh reaction solution of **4** after 1 hour heating (at ~80°C) recorded at room temperature in H_2O-D_2O mixture.

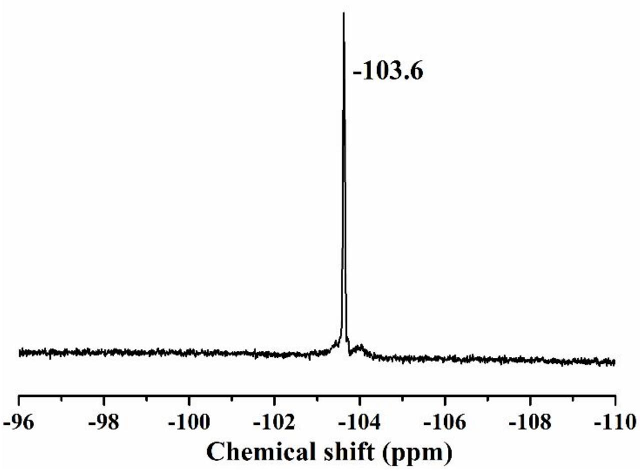

Figure S29. ^{19}F NMR of fresh reaction solution of **4** after 1 hour heating (at ~80°C) recorded at room temperature in H_2O-D_2O mixture.

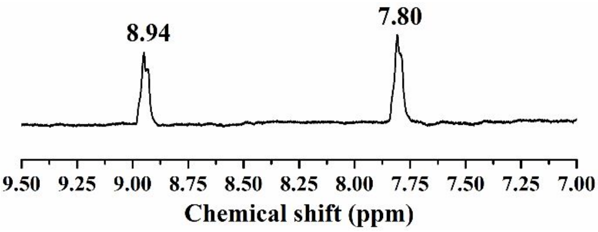

Figure S30. ^{1}H NMR of fresh reaction solution of **5** after 1 hour heating (at ~80°C) recorded at room temperature in H_2O-D_2O mixture.

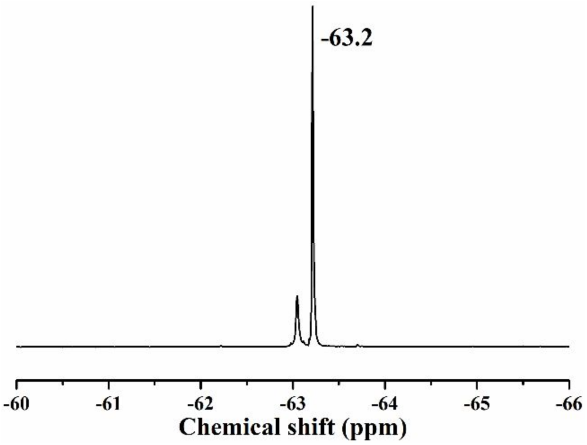

Figure S31. ^{19}F NMR of fresh reaction solution of **5** after 1 hour heating (at ~80°C) recorded at room temperature in H_2O-D_2O mixture.

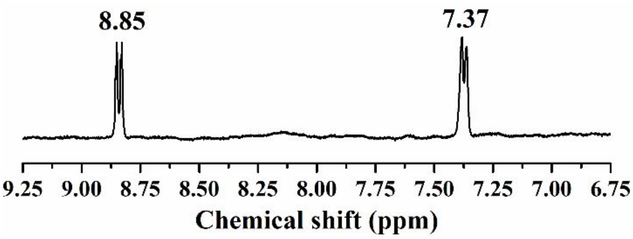

Figure S32. ^{1}H NMR of fresh reaction solution of **6** after 30 min heating (at ~80°C) recorded at room temperature in H_2O-D_2O mixture.

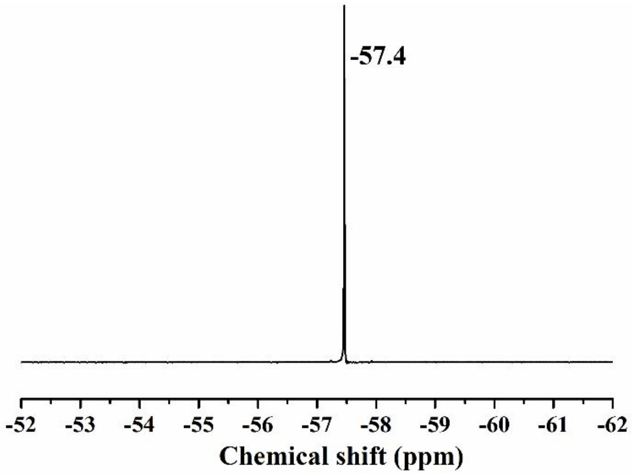

Figure S33. ^{19}F NMR of fresh reaction solution of **6** after 30 min heating (at ~80°C) recorded at room temperature in H_2O-D_2O mixture.

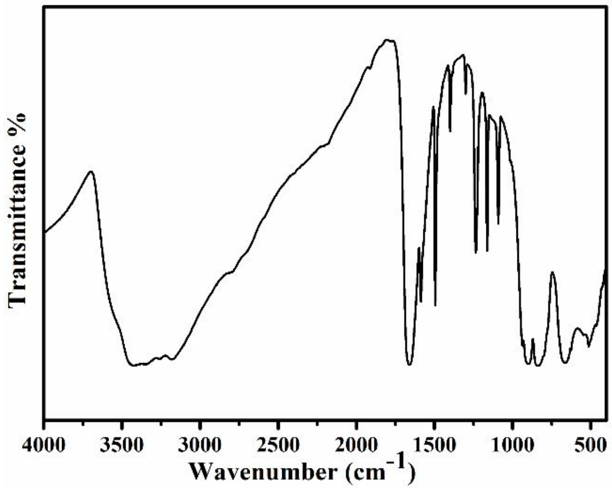

Figure S34. FTIR spectrum of **Gua-1** on a KBr disk.

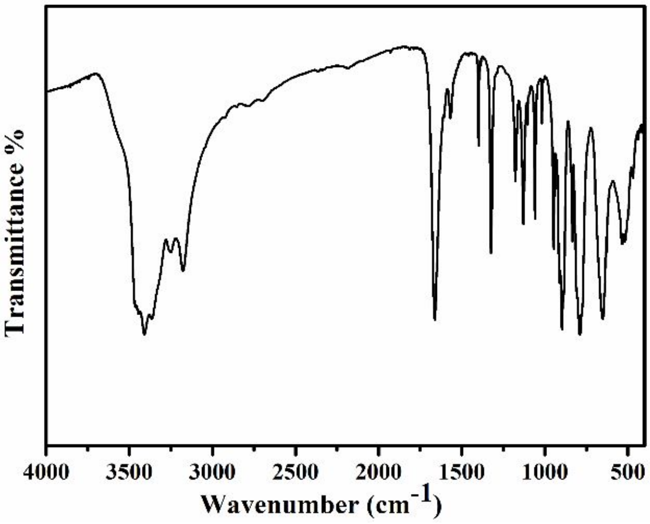

Figure S35. FTIR spectrum of **Gua-2** on a KBr disk.

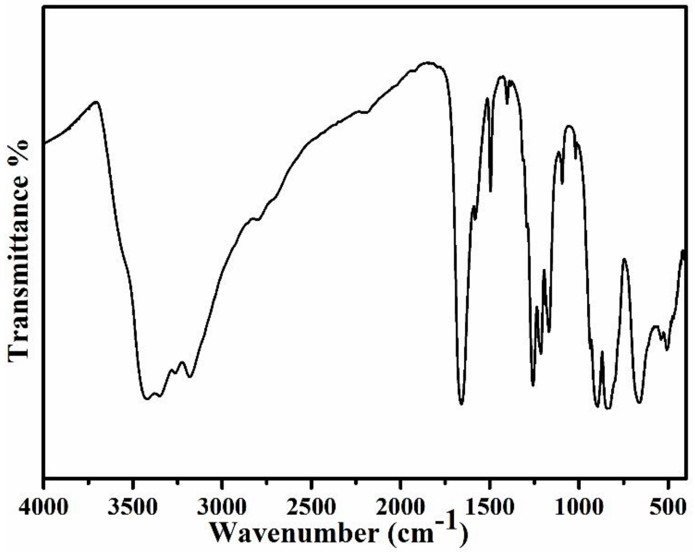

Figure S36. FTIR spectrum of **Gua-3** on a KBr disk.

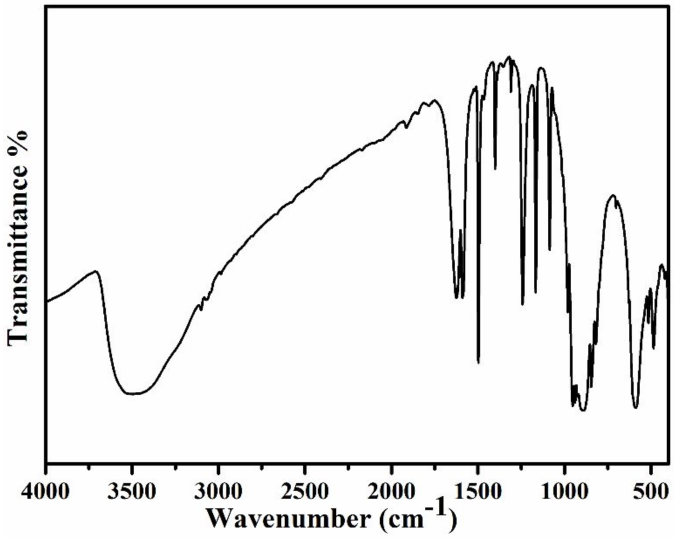

Figure S37. FTIR spectrum of **Na-4** on a KBr disk.

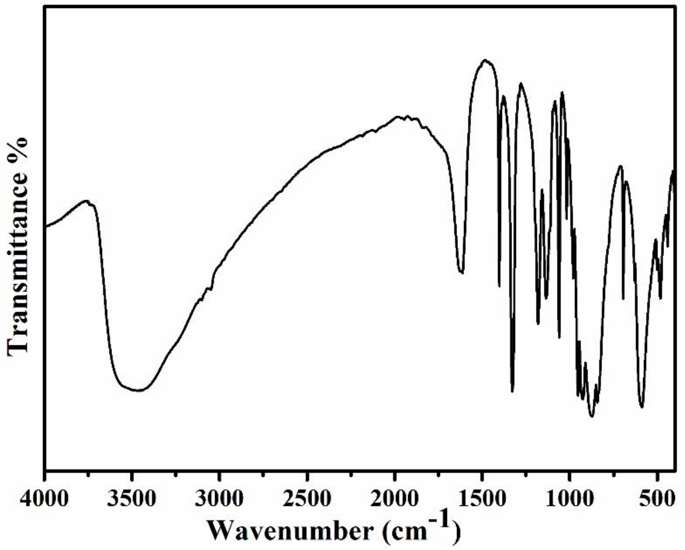

Figure S38. FTIR spectrum of **Na-5** on a KBr disk.

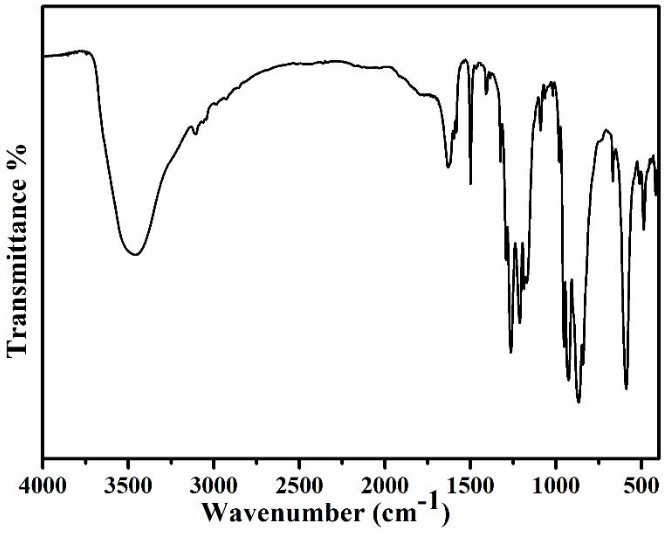

Figure S39. FTIR spectrum of **Na-6** on a KBr disk.

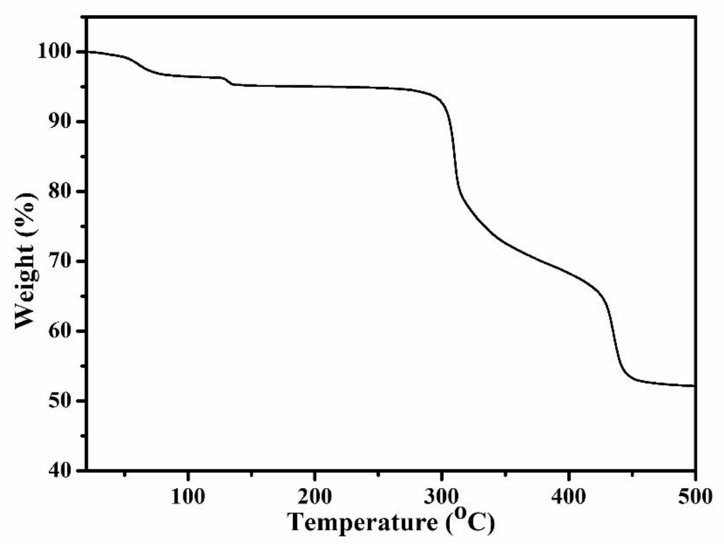

Figure S40. Thermogram of **Gua-1** from room temperature to 500 °C under N_2 flow.

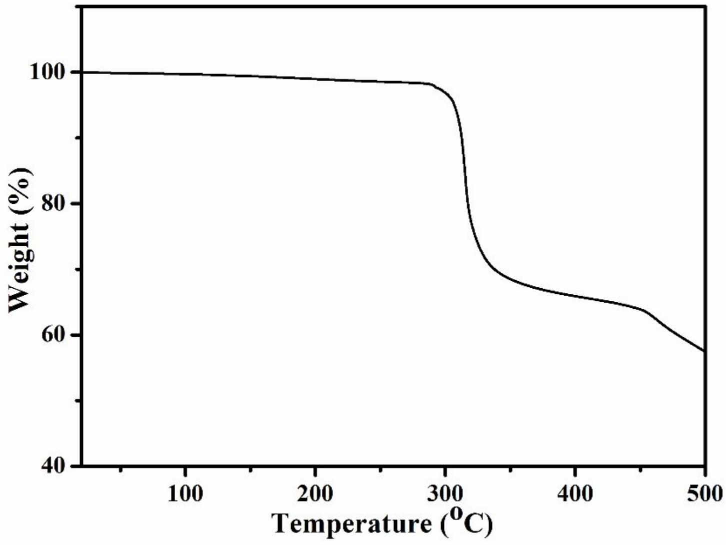

Figure S41. Thermogram of **Gua-2** from room temperature to 500 °C under N_2 flow.

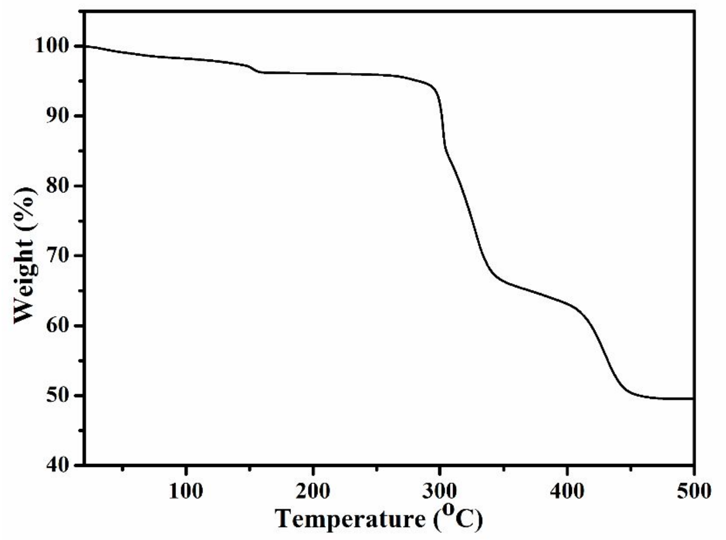

Figure S42. Thermogram of **Gua-3** from room temperature to 500 °C under N_2 flow.

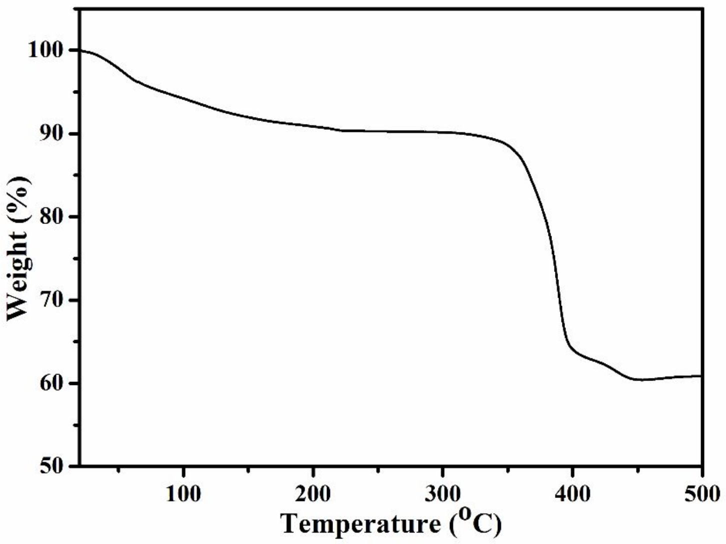

Figure S43. Thermogram of **Na-4** from room temperature to 500 °C under N_2 flow.

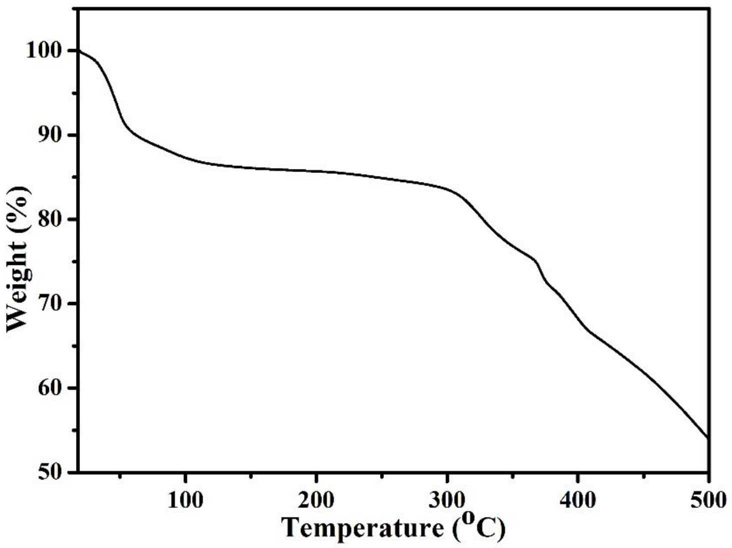

Figure S44. Thermogram of **Na-5** from room temperature to 500 °C under N_2 flow.

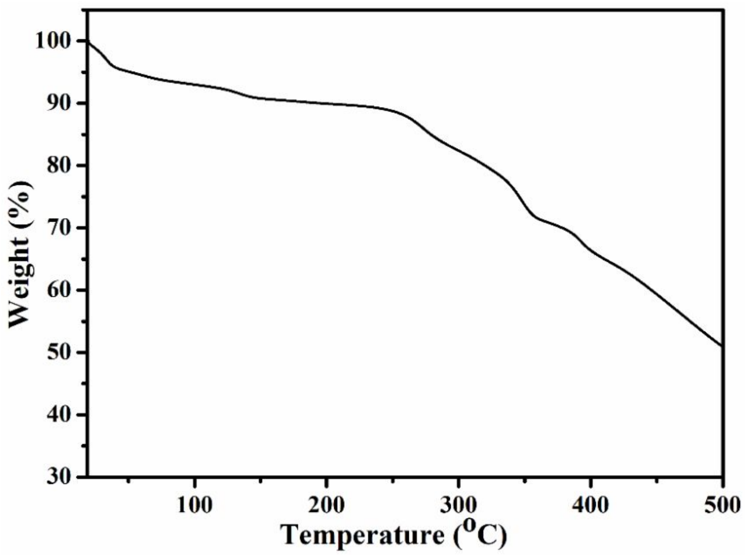

Figure S45. Thermogram of **Na-6** from room temperature to 500 °C under N_2 flow.
